# Low-intensity pulsed ultrasound promotes skeletal muscle regeneration via modulating the inflammatory immune microenvironment

**DOI:** 10.7150/ijbs.79685

**Published:** 2023-02-05

**Authors:** Haocheng Qin, Zhiwen Luo, Yaying Sun, Zhong He, Beijie Qi, Yisheng Chen, Junlong Wang, Ce Li, Weiwei Lin, Zhihua Han, Yulian Zhu

**Affiliations:** 1 Department of Rehabilitation Medicine, Huashan Hospital, Fudan University, Shanghai, China.; 2 Department of Sports Medicine, Huashan Hospital, Fudan University, Shanghai, China.; 3 Department of Orthopedics and Traumatology, Shanghai General Hospital Shanghai Jiaotong University, Shanghai, China.; 4 Department of Neurosurgery, Second Affiliated Hospital of Zhejiang University School of Medicine, Zhejiang University, Zhejiang, China.; 5 Department of Orthopedics, Shanghai Pudong Hospital, Fudan University, Shanghai, China.; 6 Department of Radiology, Huashan Hospital, Fudan University, Shanghai, China.

**Keywords:** Low-intensity pulsed ultrasound, Macrophage polarization, Inflammation, Muscle injury, WNT signaling

## Abstract

**Background:** Low-intensity pulsed ultrasound (LIPUS, a form of mechanical stimulation) can promote skeletal muscle functional repair, but a lack of mechanistic understanding of its relationship and tissue regeneration limits progress in this field. We investigated the hypothesis that specific energy levels of LIPUS mediates skeletal muscle regeneration by modulating the inflammatory microenvironment.

**Methods:** To address these gaps, LIPUS irritation was applied in *vivo* for 5 min at two different intensities (30mW/cm^2^ and 60mW/cm^2^) in next 7 consecutive days, and the treatment begun at 24h after air drop-induced contusion injury. In vitro experiments, LIPUS irritation was applied at three different intensities (30mW/cm^2^, 45mW/cm^2^, and 60mW/cm^2^) for 2 times 24h after introduction of LPS in RAW264.7. Then, we comprehensively assessed the functional and histological parameters of skeletal muscle injury in mice and the phenotype shifting in macrophages through molecular biological methods and immunofluorescence analysis both *in vivo* and *in vitro*.

**Results:** We reported that LIPUS therapy at intensity of 60mW/cm^2^ exhibited the most significant differences in functional recovery of contusion-injured muscle in mice. The comprehensive functional tests and histological analysis *in vivo* indirectly and directly proved the effectiveness of LIPUS for muscle recovery. Through biological methods and immunofluorescence analysis both *in vivo* and *in vitro*, we found that this improvement was attributable in part to the clearance of M1 macrophages populations and the increase in M2 subtypes with the change of macrophage-mediated factors. Depletion of macrophages *in vivo* eliminated the therapeutic effects of LIPUS, indicating that improvement in muscle function was the result of M2-shifted macrophage polarization. Moreover, the M2-inducing effects of LIPUS were proved partially through the WNT pathway by upregulating FZD5 expression and enhancing β-catenin nuclear translocation in macrophages both *in vitro* and *in vivo*. The inhibition and augment of WNT pathway *in vitro* further verified our results.

**Conclusion:** LIPUS at intensity of 60mW/cm^2^ could significantly promoted skeletal muscle regeneration through shifting macrophage phenotype from M1 to M2. The ability of LIPUS to direct macrophage polarization may be a beneficial target in the clinical treatment of many injuries and inflammatory diseases.

## Introduction

Skeletal muscle injury caused by traumatic accidents can impair posture and functional movement, limiting daily activities and affecting life quality 1,2. With over 20% muscle mass loss, extensive deficits need therapeutic management to support normal muscle regeneration 3. The formation and maturation of regenerating muscle fibers are critical for functional recovery, which depends on the activity of myogenic progenitor cells (MPC) or satellite cells 4. A series of immune cell infiltration and activation processes will proceed during muscle regeneration 5-7. Immune cells with specific cytokines and growth factors are involved in the clearance of damaged muscle tissue, angiogenesis, and extracellular matrix (ECM) remodeling 4,8. Therefore, it has been suggested that the emerging therapeutic approaches for muscle healing that focus on immunomodulation and immunomodulatory agents are promising in several preclinical studies. They delivered the immunomodulation medicine into injured site through direct injection 9-11. Certain materials used to fill muscle defects have been reported to modulate the immune response, thus enhancing healing 12,13. Despite the high morbidity of the donor muscle site, the surgery using autologous muscle flaps is still the standard method for patients suffering from severe muscle injuries 1,14. Therefore, an adjunctive treatment strategy without injection and invasiveness is imperative. The biological mechanisms of simple and noninvasive approaches such as the ultrasound-based method to treat skeletal muscle injuries are poorly understood, thereby limiting their clinical application 9.

Low-intensity pulsed ultrasound (LIPUS), a form of mechanical stimulation delivered through designed transducer, is widely used clinically to treat musculoskeletal soft tissue injury as an alternative and complementary medicine 15,16. Over the past few decades, there were increasing evidence showing that LIPUS therapy can reduce the expression of pro-inflammatory cytokines, limit the infiltration of inflammatory cells, and modulate the phenotype of inflammatory cells. This beneficial alteration my relate to increased blood flow, activated mitochondrial biogenesis, and anti-oxidative stress effect, thereby promoting muscle healing 17-19. Both clinical trials and preclinical research have indicated that LIPUS can reduce the inflammatory response and accelerate muscle damage recovery 20,21. However, the direct link among specific cellular and molecular components, the reduction of inflammation, and functional recovery has yet been elucidated.

In recent years, macrophage polarization has been widely studied in mediating inflammatory immune microenvironment 22-25. In general, acute muscle injury initially recruits "typically activated" macrophages (M1) into the pathological loci to perform phagocytosis of necrosis tissue and initiate myogenesis by producing nitric oxide (NO) and pro-inflammatory cytokines 26,27. Subsequently, "alternatively activated" M2 macrophages replace the M1 phenotype to promote muscle regeneration and differentiation 28,29,29. M2 subtype secretes anti-inflammatory factors, including IL-10 and TGF-β1, which shifts the inflammatory microenvironment to the inflammation-suppressed one 30. If the balance between M1 and M2 is broken, the inflammatory microenvironment will beyond control, leading to impaired tissue healing. According to our recent studies, the macrophage polarization balance between M1and M2 plays an essential role in muscle healing 31. The previous study has primarily proved that LIPUS affects the polarization state of macrophages *in vitro*, which suggested LIPUS may influence the inflammatory microenvironment through a macrophage-mediating mechanism 32,33, while it has not been clearly investigated the underlying mechanism in treating skeletal muscle injury. WNT/β-catenin signaling is essential in mediating immune microenvironment, tissue repair and regeneration 34,35. Dysregulation of β-catenin signaling is involved in persistent inflammation and organ fibrosis 36. Studies have found that WNT/β-catenin signaling promotes M2 macrophage polarization, which could further promote the resolution of inflammation 37. It was also reported that WNT/β-catenin signaling play a pivotal role in myogenesis 38. However, the role of WNT/β-catenin signaling in mediating the effect of LIPUS on inflammatory immune microenvironment in injured skeletal muscle warrants further exploration.

Several lines of evidence illustrated that LIPUS might benefit the muscle healing process 17,19,21,39. So far, the changed immune microenvironment and cellular mechanisms by which LIPUS promotes muscle healing are, however, not well understood. In the study described here, effects of LIPUS on the immune microenvironment in mouse skeletal muscle were detected and the molecular modification of macrophage polarization in an induced M1 cell model was also investigated. It was hypothesized that LIPUS enhances muscle healing by shifting the inflammatory microenvironment in a macrophage-dependent manner.

## Methods

### Establishment of Mice Contusion Model

All animal experiments were conducted under the standard of the National Institutes of Health Guide for the Care and Use of Laboratory Animals. The Fudan University Animal Care Committee authorized the experimental protocols. (Approval no.20171248A703, KY-2018-0390) and every attempt was introduced to keep animal suffering to a minimum.

In this study, a total of 104 C57BL/6 male mice (age: 10-11 weeks; weight:25 ± 3 g) obtained from Shanghai Experimental Animal Center were kept in the animal shelter facility under controlled temperature, humidity, and light, with free access to rodent food and water, and a 12-hour light/dark cycle at Department of Laboratory Animal Science, Fudan University. 72 mice were used in the normal contusion injury model, 24 mice were used in the macrophage depletion model and 8 mice were used in the negative control group for behavioral tests. A right gastrocnemius muscle contusion was induced as the muscle injury model via the dropped-weight technique. After being anesthetized and not responding to a toe pinch, the animals' hind limbs were positioned, dorsiflexing the ankle to 90°. A 16.8 g (diameter 15.9 mm) stainless steel ball was dropped from the height of 100 cm through a tube (interior diameter of tube:16 mm) onto an impactor resting with a surface of 28.26 mm^2^ on the middle of the gastrocnemius muscle of the mouse. Middle of the gastrocnemius was defined as 15 mm proximal to the calcaneus when the leg was stretched. While the left gastrocnemius (Control) was neither injured nor LIPUS treated. The instantaneous force delivered by a falling object with these characteristics was calculated to equal 0.58 N·m/cm^2^, where 1 N·m is equal to the force of an object weighing 100 g falling over 1 m. The muscle contusion model was a high-energy blunt injury that created a large hematoma and was followed by massive muscle regeneration healing processes that are very similar to those seen in humans 31. The Creatine Kinase concentrations in the serum at 2h and 24h was assessed to reflect the muscle damage in mice. No drugs (e.g., buprenorphine, NSAIDs) were given to the mice before or after the contusion injury.

After one week of acclimatization, the mice that needed to be modeled were subjected to acute muscle contusion on the right gastrocnemius muscles, with the left gastrocnemius muscles regarded as the Control muscle sample. Then, all the injured mice were randomly divided into 3 groups with 24 mice in each, named as Contusion group (n=24), Contusion+Ultrasound^L^(n=24), and Contusion+Ultrasound^H^ (n=24) respectively. We harvested muscle samples from mice on Days 3, 7, and 14. 8 mice were randomly selected from each group at each time point.

### Macrophage depletion

We established macrophage depletion mice according to the published procedure 31. In short, the mice were injected with 2 mg clodronate-containing liposomes (purchased at www. clodronateliposomes.com) intraperitoneally two days before the contusion. Then, on Day 0, 3, and 6 after muscle contusion, 0.5g of clodronate-containing liposomes were re-injected to keep the macrophages at a low level. The spleen of mice was dissected for tissue flow cytometry to identify the proportion of CD11b+F4/80+ cells. Many studies have proven that macrophages percentage in the spleen can verify the success of macrophages depletionas spleen can presented the station of immune system 40. Research have also demonstrated the fact that systemic depletion of macrophages by clodronate liposomes can deplete skeletal muscle macrophages 41,42. On Day 14, all mice with macrophage depletion had their muscle functional recovery and fibrosis assessed.

### Ultrasound treatment in mice

**Fig 1:**
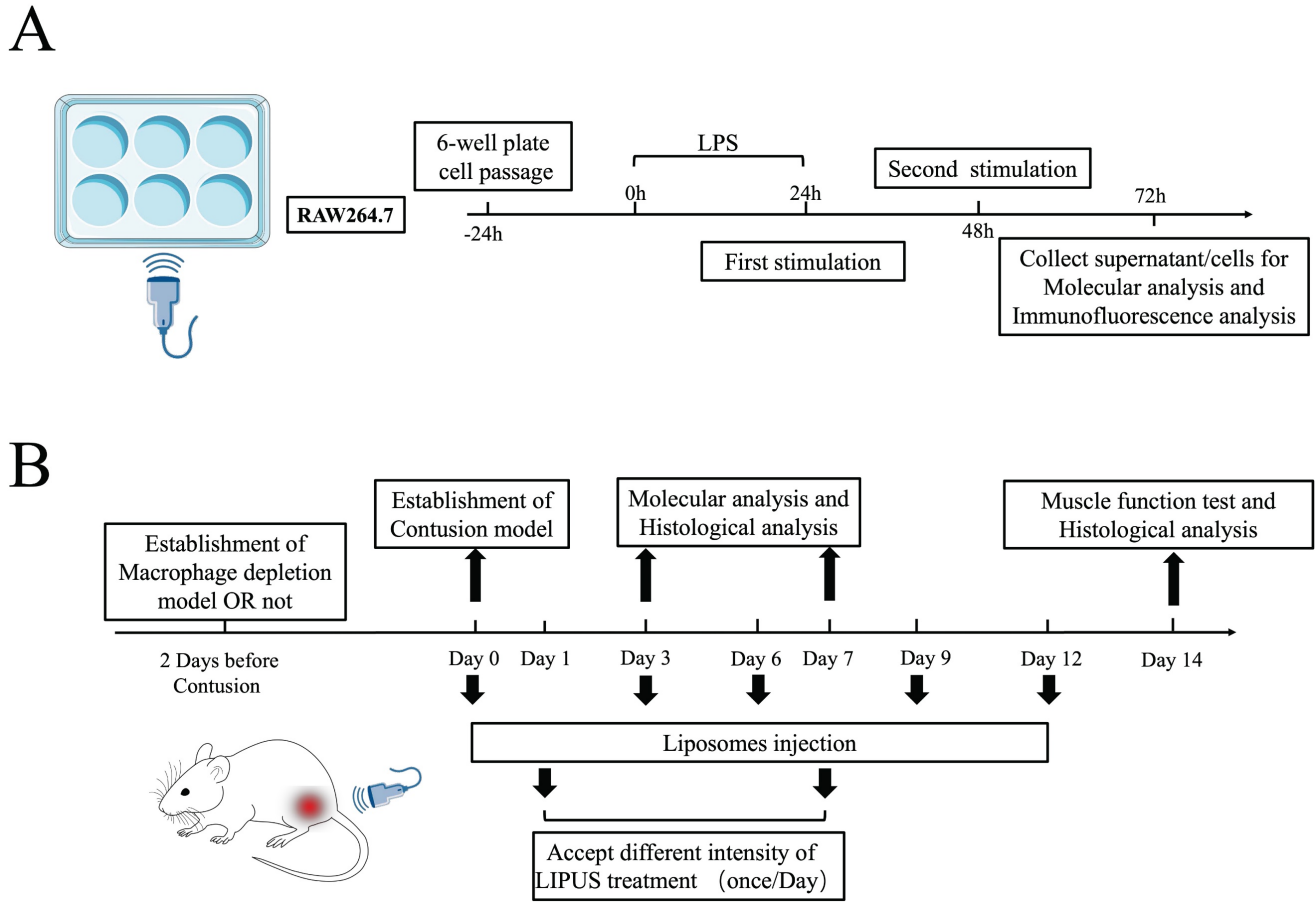
** Experimental design for both the *in vitro.* and *in vivo.* studies.** (A) Experimental design for LPS-induced RAW264.7. (B) Both the normal contusion injury model and macrophages depletion contusion were presented. Molecular Analysis included Antibody Array analysis, Western Blot, ELISA, qPCR, and flow cytometry. The histological evaluation included Laser speckle contrast analysis, HE staining, Masson staining, and immunofluorescence. Muscle function tests included the Water Maze test, Rotarod test, Treadmill test, CatWalk test, and Tetanus strength test.

Because macrophage polarization occurs primarily in the first 7 days after injury 43, mice sacrificed on Day 3 had daily treatment until Day 3, and mice sacrificed on Days 7 and 14 had daily treatment until Day 7. We applied a commercially available ultrasound gel (Jinya, China) as a coupling agent. During therapy, mice were stabilized using a restraint cage while their hind limbs were manually fixed, and the gastrocnemius area of all animals was depilated before the treatment. Before we start the research, the ultrasound equipment (Chattanooga 2776, USA) was calibrated by the Bioengineering department of Fudan University to ensure the stability and accuracy of output parameters. The treatment point was same as the drop point and the treatment begun at 24h after contusion injury. Experimental parameters are as followed. LIPUS was administered at 1 MHz frequency with a 20% duty cycle for 5 minutes using a transducer with a 2 cm^2^ effective radiating area. The intensity of the Contusion+Ultrasound^L^ and Contusion+Ultrasound^H^ groups were 0.3 W/cm^2^ and 0.6 W/cm^2^ SATP (spatial average temporal peak), respectively, while the mice in the Contusion group receive the same time treatment but with the power turned off. Notably, the 20% duty cycle (2 ms on, 8 ms off) and 2 cm^2^ effective radiating area corresponded to 30mW/cm^2^ and 60mW/cm^2^ SATA (spatial average temporal average). The procedure of the *in vivo* treatment was shown in Figure 1B, Figure S2A, and Sup Movie 1.

### Cell culture and cell intervention

RAW264.7 (mouse leukemia cells of monocyte-macrophage) were purchased from the American Type Culture Collection and constantly maintained in high glucose Dulbecco's modified eagle medium (DMEM) (HyClone) with 10% fetal bovine serum (FBS) and 0.5 ml of penicillin/streptomycin solution (#0503; ScienCell Research Laboratories). All cells were kept in an incubator at 37℃ and 5% CO_2_. The third passage of the RAW264.7 cells were used for the in vitro experiments. The cells were grown for 3 days with periodic medium changes and then seeded in 6‐well tissue culture plates. Then, 500 ng/ml lipopolysaccharides (LPS) were added to every plate to simulate the inflammatory environment (promoting M1 polarization) according to our previous study 31. Besides, the concentration we used depended on drug titration curve with cell viability shown in Fig. S7C. Six hours later, LPS was discarded and washed with PBS.

The cells were divided into four groups: LPS group, LPS+Ultrasound^L^ group, LPS+ULtrasound^M^, and LPS+Ultrasound^H^ group. Cells were LIPUS treated 24 and 48 hours after LPS was added to the culture media. Cell culture supernatant from the treated cells was collected 24 hours after the second LIPUS treatment (i.e., 72 hours after the addition of LPS) for further analysis (The details of the cell experiment are shown in Fig. 1A). The treatment parameters were the same as in mice experiments and the medium dose was set to 45mW/cm^2^. Through a coupling gel applied between the ultrasonic transducer and the plate, sound energy was passed through the bottom of the plate. The acreage of the ultrasonic transducer is the same as that of a single hole in the six-well plate. When the silence of Wnt/β-catenin signaling was required, the RAW264.7 cells were treated with XAV-939 (Wnt/β-catenin signaling inhibitor) 44 at the final concentration of 10 μM for 24h. XAV-939 stimulate β-catenin degradation through stabilizing axin 45. If the activation of WNT/signaling was required, the RAW 264.7 was treated with QS11 (Wnt/β-catenin signaling agonist) 46 at the final concentration of 10 μM for 24h treatment. The concentration we used depended on drug titration (XAV-939 and QS11) curve with cell viability shown in Fig. S10.

### Animal Sample Harvest and Analysis

Bilateral gastrocnemius muscles were dissected after the mice were euthanized. Following the isolated bilateral muscles photographed, immediately the wet weight of the muscles on both sides was measured and the ratio of the injured side to the uninjured side was calculated.

Then, strain gauge transducers coupled with a TBM4 strain gauge amplifier (World Precision Instruments Inc., Sarasota, USA) were used to test the passive force of isolated muscles from Day 14. First, we separated the lower limbs of the mice. Then, the calf bone was cut down the middle. Next, the Two bones attached to the gastrocnemius tendon were used as anchors for the test machine. A data program was used to analyze the strength (Windaq; DATAQ Instruments Inc., Akron, USA). The data was further normalized with the wet weight.

Next, the bilateral muscles were washed immediately with saline and then fixed in the 10% buffered neutral formalin solution. After 24 h, muscles were successively dehydrated in 70%, 80% (2×), 95% (2×), and 100% (3×) ethanol for 30min per step. Subsequently, they were processed with xylene (3×) for 20min per step and embedded in paraffin. Next, the injured areas of embedded muscles were cut with a paraffin slicing machine (LEICA RM2235) into 5 µm thick sections (Perpendicular to the direction of muscle fibers) for routine Hematoxylin and eosin (HE) stain, Masson stain (for fibrosis analysis), and immunofluorescence stain (for fibrosis protein).

### HE staining

Firstly, the paraffin sections from Day 3 were deparaffinized by the oven (65℃ 30min), xylene (2× 10min), and gradient alcohol (10 min per time) successively. Hematoxylin and eosin-stained were performed on the tissue separately for 10 min and 1 min. Finally, the tissue was sealed with neutral resin and observed under the microscope directly (ECHO Revolve, American). The same HE staining was performed on muscle obtained from macrophage-depleted mice on Day14. The Cross-Sectional areas (CSA) and Cellularity of muscle fibers were calculated using ImageJ software. Besides, Centronucleated cells were considered to be regenerating myofibers. Therefore, manual recording of newly produced muscle fibers was also calculated 17. The source of the samples was masked from the observer conducting the counting. All histological statistics were calculated with 4 samples included in each group. 4 unbiased (*200 magnification) images were selected from 3 section in each muscle sample.

### Masson staining

Paraffin sections from time points Day 7 and Day 14 were subjected to Masson staining. The processes for deparaffinization and rehydration were the same as for HE stains. The entire process was carried out according to the commercial kit for Masson staining (Solarbio, China) 47. The same Masson staining was performed on muscle obtained from macrophage-depleted mice. The fibrosis condition was observed and recorded by the microscope (ECHO Revolve, American). The fibrotic areas and CSA were calculated with 3 sections from 4 samples in each group. 4 unbiased (*200 magnification) images were randomly selected for targeted section. The source of the samples was masked from the observer conducting the counting.

### Immunofluorescence analysis

Paraffin sections from Day 3, and Day 14 were used for Immunofluorescence analysis. As described above, the processes for deparaffinization and rehydration were the same as for HE staining. Then, the water surrounding the tissues was cleaned up, and a fluorescent pen was used to draw circles around the tissues on each slide. Then, 5% BSA and 0.5% Triton-X-100 (Solarbio, Beijing, China) were used to block the tissue at RT for 1h and diluted primary antibodies were used to incubate with muscle overnight at 4°C. On the second day, 1× PBST washing for 5 min three times was performed before and after incubation with Alexa Fluor 488 anti-mouse (H+L) secondary antibodies (1:500; Life Technologies, USA) for 1 hour at RT. DAPI was used to locate nuclei. As for cell immunofluorescence, the DMEM was removed in the first step. Then after being rinsed with PBS, cells were fixed in 4% PFA for 10 min and were counterstained cytoskeleton with Phalloidin-594 for 30 min. In the procedures of permeabilization and blockage, 5% BSA with 0.5% Triton-X-100 was used for 70 min at RT. Next, immunofluorescence was incubated with primary antibodies incubation at 4°C overnight (1:100 dilution for β-catenin [ab32572; Abcam]. 1:200 dilution for iNOS [AF0199; Affinity], CD86 [ab64693; Abcam], CD206 [ab64693; Abcam], CD68[ab955; Abcam]. 1:500 dilution for FZD5 [ab7523; Abcam], F4/80[ab90247; Abcam].) and then with the corresponding secondary antibody for 1 hour at RT. Finally, DAPI counterstained the nuclei. Images were observed by a fluorescence microscope (ECHO Revolve, America). All images taken used the same microscope settings (e.g., exposure time, laser intensity, and gain).

### *Flow cytometry for M1/M2* in vitro and macrophages in vivo

The Cytomics™ FC 500 (Beckman Coulter) was used to calculate the targeted cells by the surface markers of macrophages, including anti-CD86-PE, anti-CD163-APC, anti-CD11b-FITC, anti-F4/80 APC, and anti-F4/80-FITC (Thermo/eBio) 28,48. Firstly, macrophages were collected with flow cytometry staining buffer (eBioscience). After adding 2ul of antibody to every 100ul of cell suspension and incubating for 60 min at 4°C in the dark, 5 mL staining buffer was added into each tube and the cell suspension was centrifuged for 5 min (500 × g, 4 ℃). Followed by three times of repeated washing procedures, the cell was re-suspended in 200 μL PBS for final flow cytometry analysis. In experiment of validation for macrophages depletion in mice, the spleens from the mice treated with clodronate-containing liposomes were surgically removed on Days 1, 3, and 7 after injection. Spleen from control was also collected. Dispase, Collagenase, and trypsin were used to digest the tissue matrix and isolate the cells. The cell suspension was used for final flow cytometry analysis 31.

### Water maze

**Fig 2:**
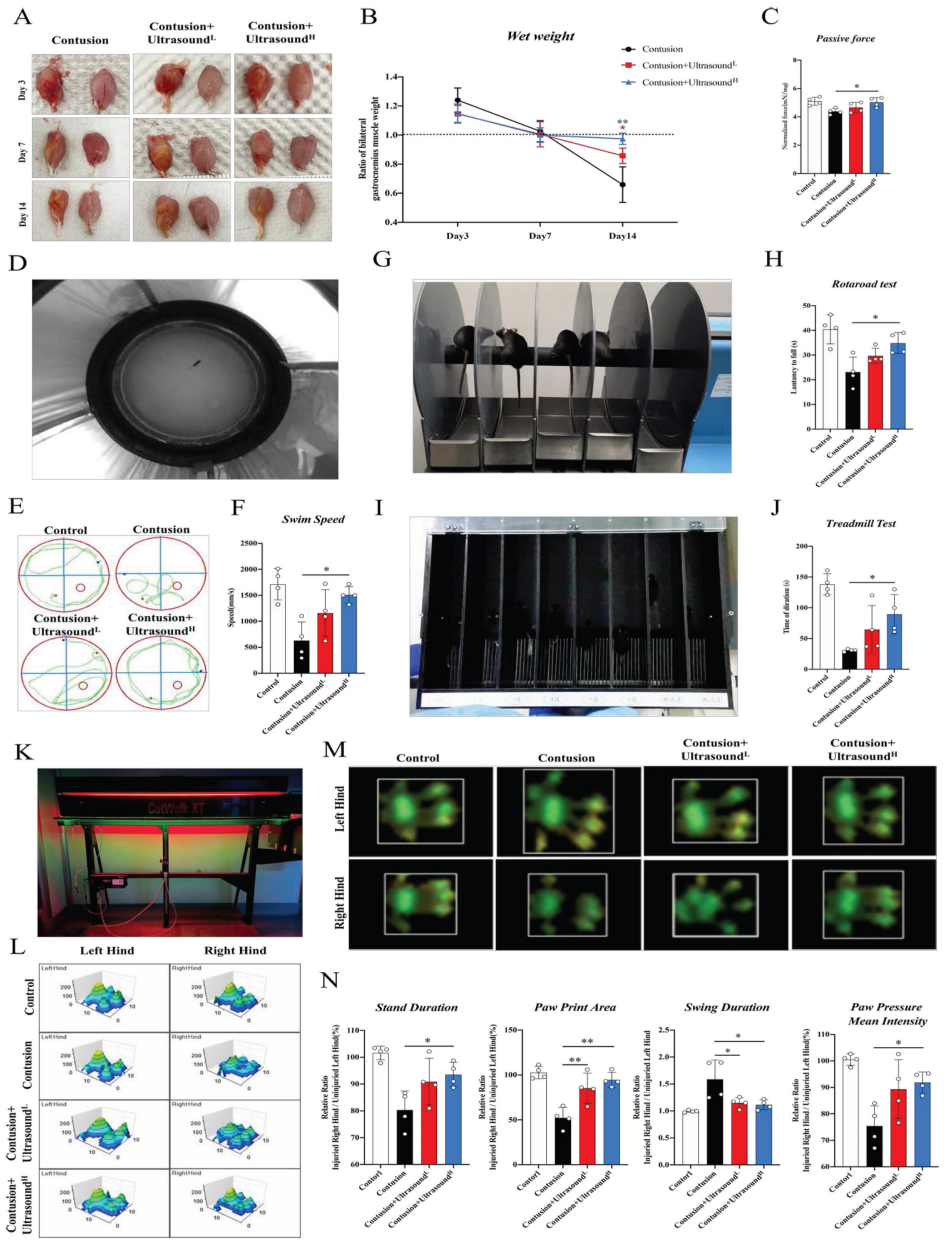
** LIPUS promoted muscle functional recovery.** (A-C) gastrocnemius muscles were directly observed on Day 3 after dissecting. The ratio of bilateral gastrocnemius muscles weight at different time points. (n=4) Red*P<0.05(Contusion+Ultrasound^L^ group compared with Control group). Bule**P<0.01(Contusion +Ultrasound^H^ group compared with Control group). Statistics of passive strength of isolated gastrocnemius muscles. (n=4) *P<0.05. (D-F) A photo was recorded during the Water Maze test and a representation of the movement trajectories of mice over 1 minute in different four groups. The swimming speed of mice in 1 minute was compared. (n=4) *P<0.05. (G-H) Photos were taken during the Rotarod test. The duration time of mice on the accelerated rod was recorded and compared among different groups. (n=4) *P<0.05. (I-J) Photos were taken during the Treadmill test. From left to right, there are two mice from the Control group, two mice from the Contusion group, two mice in the Contusion +Ultrasound^L^ group, and two mice from the Contusion +Ultrasound^H^ group. (n=4) *P<0.05.**P<0.01. The duration time till fatigue of mice on the acceleration band was recorded and compared among different groups (n=4) *P<0.05. (K-L) A photograph of the apparatus used to conduct the gait experiment and representative images of three-dimensional paw pressure distribution of mice in different groups. Quantitative analysis of four gait parameters of hind limbs, including the ratio of bilateral swing time, bilateral standing time, bilateral paw pressure mean intensity, and bilateral paw print area. (n=4) *P<0.05.**P<0.01.

The water maze was utilized to acquire the swim speed of mice, which was regarded as an indicator of recovery of locomotion ability 49. In brief, when the mice were put into the water, the timing was started, and the distance the mice swam was recorded for the next 60 seconds to obtain the average speed of movement (Fig. 2D). The swimming patterns were recorded by the Video Tracking System (ActualTrack™).

### Treadmill test

Exercise capacity was also determined using a treadmill (Anhui Zhenghua Biologic Apparatus Facilities, China) running tests 50. The mice were acclimated to the treadmill for 10 min with a slope of 5% and speed of 10m/min for 2 days before the test. 6 shocks in 30 seconds were defined as fatigue and the intensity of the shock is set to 0.6mA 51. The initial speed of the platform is set at 10 m/min. Before the experiment, all mice were allowed to jog at this speed for 2 min. After that, the platform accelerated at 1 (m/min)/s until it reached 25m/min (Fig. 2I). After each test, the mice were given a 30-min rest period to minimize the effect of different test running time of each mouse, and the three tests were completed in the same day. Four mice from each group were randomly selected in this experiment.

### Catwalk test

CatWalk XT™ system (Noldus Information Technology, Netherlands), a fully automated and highly sensitive instrument for assessing voluntary movement and gait, was applied for gait analysis of mice (Fig. 2K). 4 mice in each group were randomly selected for the experiment on Day 14. Similar to a human clinical gait test, the system allows rodents to move autonomously within a restricted detection channel. A green LED light was emitted into the glass board, and high-speed digital cameras 30 cm away recorded the paw prints in real-time refracted by every contact with the glass. The intensity of the reflected light is proportional to the pressure placed on the glass. Before each testing session, mice were habituated to the testing room for 30 min and then mice were acclimated to the tunnel for 5 entire passes. The system was cleaned between each mouse tested. For each animal, three successful runs were acquired and recorded. We defined successful runs as spending longer than 2.0 s, but shorter than 10.0 s (5 gait cycles on average), with a maximum allowed speed variation ≤ 40%. Runs were rejected if the animal turned around.

### Rotarod test

Animals were briefly pre-trained on an automated 5-lane rotarod unit (Rotarod for mice; Ugo Basile, Italy, 3 cm diameter drums which are suitably machined to provide grip. Five flanges divide the five 5.7cm lanes, enabling five mice to be simultaneously on test.) that could be set on fixed speed or accelerating speed. The mice were trained for two days with five attempts each day with a rest of 20 min. 4 mice in each group were randomly selected from each group and then placed on a rod that accelerated smoothly from 5 to 40 rpm over a period of 5 min. Each mouse was tested three times with a rest of 20 min between each trial. The length of time that each animal was able to stay on the rod was recorded as the latency to fall, registered automatically by a trip switch under the floor of each rotating drum (Fig. 2G). If the mice were out of control and made three passive turns, the timing was also stopped.

### Real-time quantitative PCR (qPCR)

RNA expression of both in vivo and in vitro experiments was analyzed as previously reported 48. Total RNA was isolated by the Trizol reagent (Invitrogen, Carlsbad, CA) according to the manufacturer's instructions. RNA sample quantities were verified using a Nanodrop 2000 Spectrophotometer (Thermo Fisher Scientific, Waltham, United States) and the excessive concentration of RNA was diluted in appropriate proportions to achieve a final concentration of 200ng/μl. RNA was determined to be of good quality based on A260/A280 values (>1.8). RNA was then reversely transcribed by the PrimeScript RT reagent kit (Takara Bio). The operation was performed on the ABI7900 Real-Time PCR System (Applied Biosystems) with gene-specific primers listed in the table (Table 1). All samples were run in triplicate. The 2-ΔΔCT approach was used to determine the relative changes in gene expression of CD86, iNOS, CD206, and Arg1, which were finally normalized against GAPDH and Control samples.

The primers for PCR were as follows:CD86 forward, 5'-TGACCGTTGTGTGTGTTCTGGA-3', CD86 reverse, 5'-TCTCTGTCAGCGTTACTATCCCG3', CD206 forward, 5'GCTGGCGAGCATCAAGAGTA-3', CD206 reverse, 5'-AGGAAACGGGAGAACCATCAC-3', Arg1 forward, 5'-CATATCTGCCAAAGACATCGTG-3', Arg1 reverse, 5'-GACATCAAAGCTCAGGTGAATC-3' iNOSforward, 5'-GGGCTGTCACGGAGATCAATG-3'iNOSreverse, 5'-GCCCGGTACTCATTCTGCATG-3', GAPDH forward, 5'-CCTCGTCCCGTAGACAAAATG-3', GAPDH reverse, 5'-TGAGGTCAATGAAGGGGTCGT-3'.

### Enzyme-linked immunosorbent assay (ELISA)

The ELISA kits purchased from Laizee (LEM060-2, LEM100-2, LEM822-2, LEM810-2) were used to analyze the changes in inflammatory cytokines of cell experiments. After culture-supernatants were collected and concentrations of inflammatory cytokines IL-10, IL-6, IL-1α, and TNF-α were measured according to the manufacturer's instructions, respectively.

### Protein preparation and western blotting analysis

Among the mice sacrificed on Day 3 and Day 7, we randomly selected 4 mice from each group for protein analysis. The gastrocnemius muscle on the injured side of each mouse was dissected and collected. Tissue samples were lysed with protein extracts containing RIPA lysis buffer (Beyotime, China) and protease inhibitors (Beyotime, China). Protein was extracted from RAW264.7 also using RIPA lysis buffer (Beyotime, China). Protein from both sources was quantified using the Bicinchoninic Acid (BCA) Protein Assay Kit (Pierce, Appleton, WI, USA). Final protein loading concentration was controlled to 2.5µg/µL. When protein analysis of nuclear translocation was needed, Nuclear and Cytoplasmic Protein Extraction Kit (Beyotime, China) was used to separate the nuclear protein and cytoplasmic protein in cells of tissues and macrophages, and the whole operation was conducted according to the manufacturer's instruction. The next procedures for the protein analysis are the same. 10 % sodium dodecyl sulfate-polyacrylamide gel electrophoresis (10 % SDS-PAGE) was used to separate an equal quantity of protein, which was subsequently deposited onto PVDF membranes (Millipore, Billerica, MA, USA) at 400mA for 1 h with a cold pack. After that, the membranes were incubated for 1 hour at room temperature (RT) in a 5 percent bovine serum albumin blocking solution. Next, the primary antibody was incubated overnight at 4℃ with an appropriate primary antibody (1:1000 dilution for iNOS [AF0199; Affinity], Arg1[DF6657; Affinity], CD86 [ab64693; Abcam], β-catenin [ab32572; Abcam], FZD5 [ab75234; Abcam]. 1:2000 dilution for LaminB1[ab16048; Abcam], CD206 [ab64693; Abcam]. 1:5000 dilution for GAPDH [ab8245; Abcam], β-Tubulin [ab52623; Abcam]). The next day, the secondary antibody was incubated at RT for 1h after 3 required washing procedures. Finally, the ECL luminescence solution was used to expose the target protein (Biosharp, China). Anti-CD86, anti-CD206, anti-Arginase 1, anti-iNOS, anti-β-Tubulin, anti-FZD5 anti-β-catenin, anti-GAPDH, and anti-laminB1 were used as primary antibodies (Table 2). Each group at different points contained 4 protein samples for calculation (n=4/group).

### Laser speckle contrast analysis (LASCA)

Real-time blood perfusion to the gastrocnemius muscle was measured with a blood perfusion imager (PeriCam PSI System, Perimed AB, Stockholm, Sweden) based on Laser Speckle Contrast Analysis (LASCA) technology. Firstly, to facilitate measurement and remove the effect of fur, the mice were anesthetized and shaved at the aimed area. Then, mice were placed in a supine position with the camera aimed at the medial side of the gastrocnemius muscle. A constant-temperature plate was used to keep the mice warm. When the body temperature reached 37±0.5 ℃, we recorded the average distribution of blood perfusion for 2 min in real-time by PSI scanning. We used a 40 mm^2^ circle to determine the exact location of the muscle in each picture so that accuracy of the results can be improved. The software helped us automatically output the average perfusion amount.

### Mouse Inflammation Antibody Array

Mouse Inflammation Antibody Array (ab133999, Abcam) with 40 inflammatory targets was used to evaluate the comprehensive effect of ultrasound on the inflammatory microenvironment after muscle injury in mice. The entire experiment operation process strictly follows the manufacturer's instructions. Briefly, Antibody array membranes were blocked for 1 hour in 2 ml of blocking solution, then incubated overnight at 4 °C with 2 mL of samples and antibody mixtures. After discarding the samples, three washing procedures were performed at RT. Following the washing, the membranes were then incubated in 1:1000 diluted streptavidin-horseradish peroxidase at RT for 1h. Before the Chemiluminescent detection, membranes were washed thoroughly. Finally, the membranes were imaged.

### Cell counting kit-8 (CCK-8)

Cell counting kit-8 assay (CCK-8, Beyotime Biotechnology, Shanghai, China) was performed to evaluate the viabilities of RAW macrophages after different interventions 28. After treatment of ultrasound on a 6-well plate, the RAW 264.7 cells (1×10^3^ cells/well) were seeded in a 96-well culture plate. 4 replicate wells were set in each group. 2 μl of CCK8 reagent (Bio-Rad, Hercules, CA, USA) was added to each well, incubated at 37° C for 2 h. The absorbance was measured at 450 nm.

### Statistical analysis

All experiments were performed three technical replicates. Data were analyzed with GraphPad Prism 9.0 (GraphPad Software, La Jolla, USA) and were presented by mean ± SD. Significance was typically analyzed by Student's t-test, one-way ANOVA followed by post hoc LSD test, and two-way ANOVA followed by multiple t-tests. P < 0.05 was regarded as significant.

## Results

### Experimental model for mouse contusion injury and LIPUS setting for mouse/macrophage

After modeling, the skin of the targeted tissue was intact, and no fracture was found on the tibia and fibula bones. The introduction of a repeatable contusion-induced device ensured the standardization of the mouse gastrocnemius contusion model. Creatine kinase (CK) expression measurement was randomly administrated in 4 mice before, 2h after, and 24h after operation respectively and the serum index showed that it raised 8 to 12-fold at 2h post-injury and restore 24h later, which indicated the success of our model (Fig. S1).

In the preliminary experiment, we mainly explored the LIPUS stimulus dose. With other parameters unchanged (20% duty cycle, 1MHz, 5min), 30 and 60 (mW/cm^2^) output energy could significantly reduce the fibrosis of muscle tissue compared to 10, 20, and 90 (mW/cm^2^) (Fig. S7C-D). Combined with the other articles, these two output energies were included as the only variables. After conversion, the intensity was from 1/3 to three-folds of the clinical dose.

As for LIPUS intensity for cell experiments, the CCK8 assays were utilized to evaluate the effect of LIPUS on cell activity. The result suggested the intensity above 75 (mW/cm^2^) attenuated the activity of RAW 264.7 cells (Fig. S7A). Referring to parameters in *vivo*, the treatment protocols *in vitro* were finally decided to perform with gradient intensity of 30, 45, 60 (mW/cm^2^).

The reason why we chose Day 3 as our first observation time point is that the polarization of macrophages reached its peak 72h after injury 52. So, the first 72h is the critical period for intervention strategy to regulate macrophage polarization. Accordingly, the inflammatory activity basically ended around the Day 7, so the Day 7 was chosen to observe the final inflammation status of the injury site 43. Besides, according to our experience in previous experiments, by the Day 14, the fibrosis of muscle injury has been basically formed. Therefore, we chose day 14 as our final observation point.

### LIPUS improves functional performance in injured skeletal muscle

We first analyzed the hematoma of injured gastrocnemius muscle collected from Day 3 in different groups. In an intuitive aspect, the serious hematoma in the Contusion group was observed, while the condition was mitigated in the LIPUS-intervened groups. At the following time points, the muscle samples collected in the Contusion group exhibited atrophy, while LIPUS prevented this pathological transformation, rendering muscles fuller (Fig. 2A). To further explore the potentially affected properties of skeletal muscle brought by ultrasound, wet-weight measurement, which is one of the indications of edema, was performed. Results showed an increasing trend in the Contusion group on Day 3, when normalized to the contralateral side and compared to other groups, which indicated ultrasound could relieve early edema. In addition, on day 14, a significant increase in muscle wet was observed in the high-level ultrasound treatment group compared with the contusion group which suggested that ultrasound can prevent atrophy caused by traumatic injury (Fig. 2B). The benign effect of LIPUS in the acute stage and weight returned in the subacute stage suggested its modulation in inflammation retreating and muscle regeneration. In addition, isolated muscles from the Contusion+Ultrasound^H^ groups possessed better passive mechanical properties (Fig. 2C).

In order to dig and comprehend the effect of LIPUS on recovery of muscle function deeply, we conducted several commonly used behavioral experiments on day 14 (Fig D, G, I, K display the behavioral test included). In the water maze test, the treatment session considerably improved the swimming patterns and speed in Contusion+Ultrasound^H^ groups (Fig. 2E-F and Sup Movie 2), which revealed a fact that the recovery of locomotion ability was improved by LIPUS. The two tests-the Rotarod test and the Treadmill test represented coordination and motor persistence in mice respectively. Experimental results also indicated the injury-amelioration following LIPUS, which was more pronounced in the Contusion+Ultrasound^H^ group (Figure G-H and I-J, presented in Sup Movies 4 and 5). Last, gait analyses of voluntary locomotion of mice were recorded via the CatWalk XT system. The 3D pressure distribution images and green intensity paw print exported from the system showed a visible improvement in paw print intensity in the Contusion+Ultrasound^H^ group (Fig. 2 L-M), which was consistent with subsequent paw print mean intensity analysis (Fig. 2N). Besides, Contusion+Ultrasound^H^ group exhibited significantly improved performance on three other gait parameters including stand duration, paw print area, and swing duration, while paw print area and swing duration was also significantly improved in Contusion+Ultrasound^L^ group, when compared to the Contusion group. (Fig. 2N, shown in Sup Movie 3, detailed gait patterns were shown in Fig. S2B). These findings suggested that a specific range of therapeutic ultrasound (Contusion+Ultrasound^H^) applied on early-stage comprehensively promoted functional recovery of severely impaired muscle, including increased wet weight, passive muscle mechanics, and functional assessments.

### LIPUS improved skeletal muscle recovery in the histological aspect

**Fig 3:**
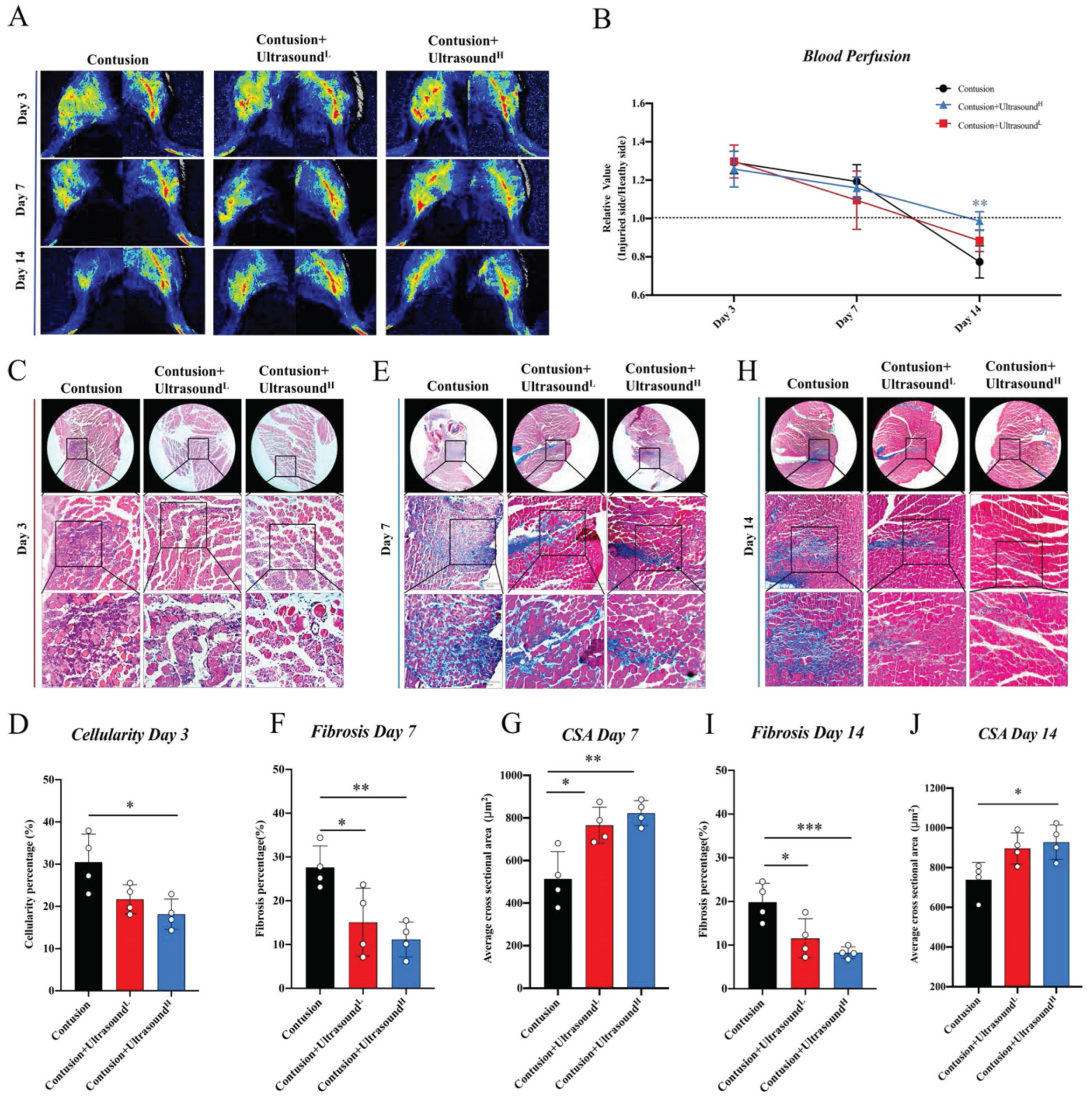
** LIPUS relieved inflammation, enhanced angiogenesis, inhibited fibrosis, and improved myogenesis *in vivo.*** (A-B) Representative blood perfusion images of bilateral gastrocnemius muscle. Circle marked the aimed injured area. The ratio of blood perfusion (injured/uninjured) of lower limbs in different groups was calculated and compared vertically and horizontally. (n=4) Bule**P<0.01(Contusion +ultrasound^H^ group compared with Control group). (C-D) Representative HE staining images of injured muscle among different groups on Day 3. The scale bar from top to bottom is 200μm. Cellularity percentage on Day 3 was calculated in different groups. (n=4) *P<0.05. The scale bar from top to bottom is 250μm, 100μm, and 50μm. (E-J) Representative Masson staining images of injured muscle among different groups on Day 7 and Day14. The scale bar from top to bottom is 250μm, 100μm, and 50μm. Fibrosis percentage on Day 7 and Day 14 among different groups was compared. (n=4) *P<0.05 **P<0.01 ***P<0.001. CSA on Day 7 and Day 14 among different groups was compared. (n=4) *P<0.05 **P<0.01.

The subsequent experiments were utilized to further explore the improvement result from ultrasound in the histological aspect. By comparing the blood perfusion in the gastrocnemius region among three groups, LIPUS was found to lead to more physiological blood perfusion on Day 14 (Fig. 3A-B, the representative parameters of the Contusion group were shown in Fig. S4A-B), which, to some extent, hints association between LIPUS and angiogenesis. By analyzing HE staining of muscles harvested on Day 3, we found that the cell infiltration was increased in the Contusion group, accompanied by a large area of necrotic muscle fibers, while the situation was alleviated in the Contusion+Ultrasound^L^ and Contusion+Ultrasound^H^ group (Fig. 3C-D). By analyzing Masson staining of muscles harvested on Day 7 and Day 14, we observed the massive interstitial fibrosis in the Contusion group, which, however, turned out to be more mitigatory in Contusion+Ultrasound^L^ and Contusion+Ultrasound^H^ group (Fig. 3E, 3F, 3H, 3I, and S3). The CSA in Contusion+Ultrasound^L^ and Contusion+Ultrasound^H^ group was also significantly improved on Day 7 (Fig. 3G). However Only CSA in Contusion+Ultrasound^H^ group was significantly improved. These conclusions versatilely confirmed the therapeutic effect of LIPUS on impaired muscle in different periods and Contusion+Ultrasound^H^ exhibited better effect in blood perfusion recovery, fibrosis reversal, decreased cellularity infiltration, and muscle regeneration.

### LIPUS modulates the inflammatory immune microenvironment through shifting macrophage polarization

**Fig 4:**
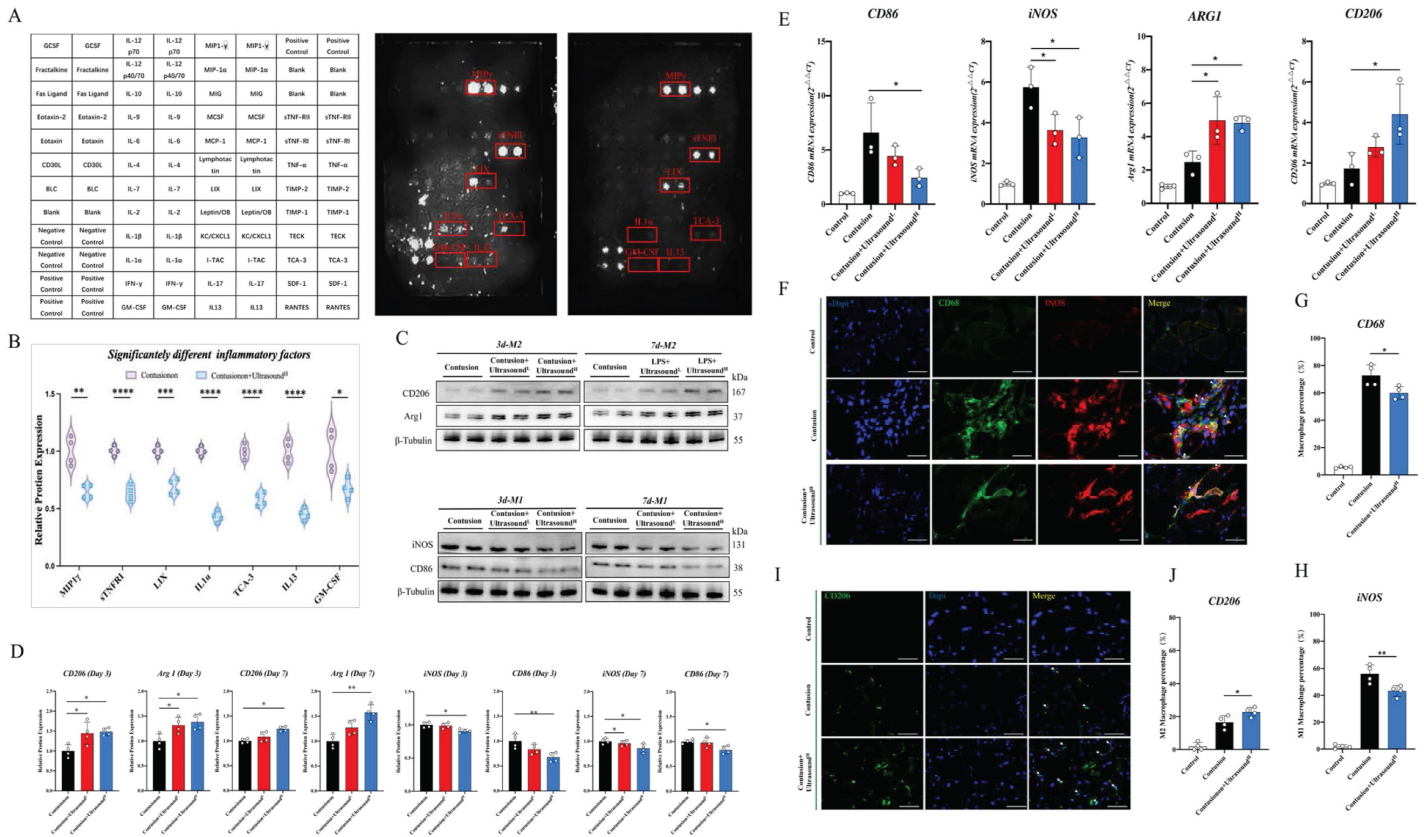
** LIPUS regulated macrophage polarization and inhibited the inflammatory microenvironment *in vivo.*** (A-B) A diagram to show the location of all the cytokines on the array. The original images showed significantly decreased antibodies which were also highlighted with a red box. Relative protein expression of significantly changed inflammatory cytokines after LIUPS treatment. (n=4) *P<0.05 **P<0.01 ***P<0.001 ****P<0.0001. (C-D) The expression level of CD206, Arg 1, iNOS, and CD86 in injured gastrocnemius muscles from different groups on Day 3 and Day7 were determined by Western Blot. (n=4) *P<0.05 **P<0.01. (E) The gene expression levels of CD86, iNOS, ARG1, and CD206 in injured gastrocnemius muscles from different groups on Day 3 were detected by qPCR. (n=3) *P<0.05 **P<0.01. (F-H) Immunofluorescence was utilized to measure the relative expression level and distribution of CD68(green) and iNOS(red) among the three groups on Day 3, including the Control group, the Contusion group, and the Contusion +Ultrasound^H^ group. The nuclei were dyed with Dapi (blue). Arrows indicate iNOS positive and CD68 positive cells. The percentage of CD68 (macrophage marker) positive cells were counted. The percentage of both CD68 and iNOS positive cells was counted. (n=4) *P<0.05. (I-J) Immunofluorescence was used to detect the relative expression and distribution of CD206(green) among the three groups on Day 3, including the Control group, the Contusion group, and the Contusion +Ultrasound^H^ group. The nuclei were dyed with Dapi (blue). Arrows indicate CD206 positive cells. The percentage of CD206 positive cells was counted. (n=4) *P<0.05.

To determine the specific microenvironment modulation evoked by LIPUS, an inflammation array was applied to investigate its impact on the acute phase of muscle injury, and a total of 44 pro-inflammation-associated factors were examined (Fig. 4A). The array test assisted us to seek out 7 significantly down-regulated factors (Fig. 4B).

Factors did not decrease significantly shown in Fig. S5. These down-regulated 7 factors consisted of 5 inflammatory chemotactic factors, including MIP1γ, LIX, TCA-3, IL-13, and GM-CSF, as well as an inflammatory receptor, sTNFRI, and a macrophage M1 polarization-promoting factor-IL-1α. Therefore, we hypothesized that LIPUS-mediated regulation of the immune microenvironment may be associated with macrophage status. Then, macrophage polarization-related parameters of two types of macrophages were confirmed in injured muscle collected on Day 3 and Day 7 after daily LIPUS intervention. After analyzing the polarization-associated proteins on Day 3, the data revealed that the M2-related proteins (Arg1 and CD206) in Contusion+Ultrasound^L^ and Contusion+Ultrasound^H^ group were significantly higher than those in the Contusion group, while only the Contusion+Ultrasound^H^ group significantly down-regulated M1-related proteins (CD86 and iNOS) (Fig. 4C-D). The proteins from Day 7 revealed that M2-related proteins (Arg1 and CD206) were significantly increased in Contusion+Ultrasound^H^ group, while the proteins expression of iNOS was significantly decreased in both Contusion+Ultrasound^L^ and Contusion+Ultrasound^H^ group, and the proteins expression of CD86 was only decresed in Contusion+Ultrasound^H^ group. The genes expression analysis on Day 3 collected muscle revealed that Contusion+Ultrasound^H^ group not only inhibited the genes expression of CD86 and iNOS but also promoted the genes expression of Arg1 and CD206. Contusion+Ultrasound^L^ group suppressed the genes expression iNOS and activated the genes expression of Arg1(Fig. 4E). In addition, immunofluorescence staining of muscle tissue harvested on Day 3 presented the same results which are that Contusion+Ultrasound^H^ group reduced the recruitment of macrophages in tissue and promoted the arrived macrophages to an anti-inflammatory state (more CD206 positive cells were presented in the ultrasound-treated group) (Fig. 4F-J).

**Fig 5:**
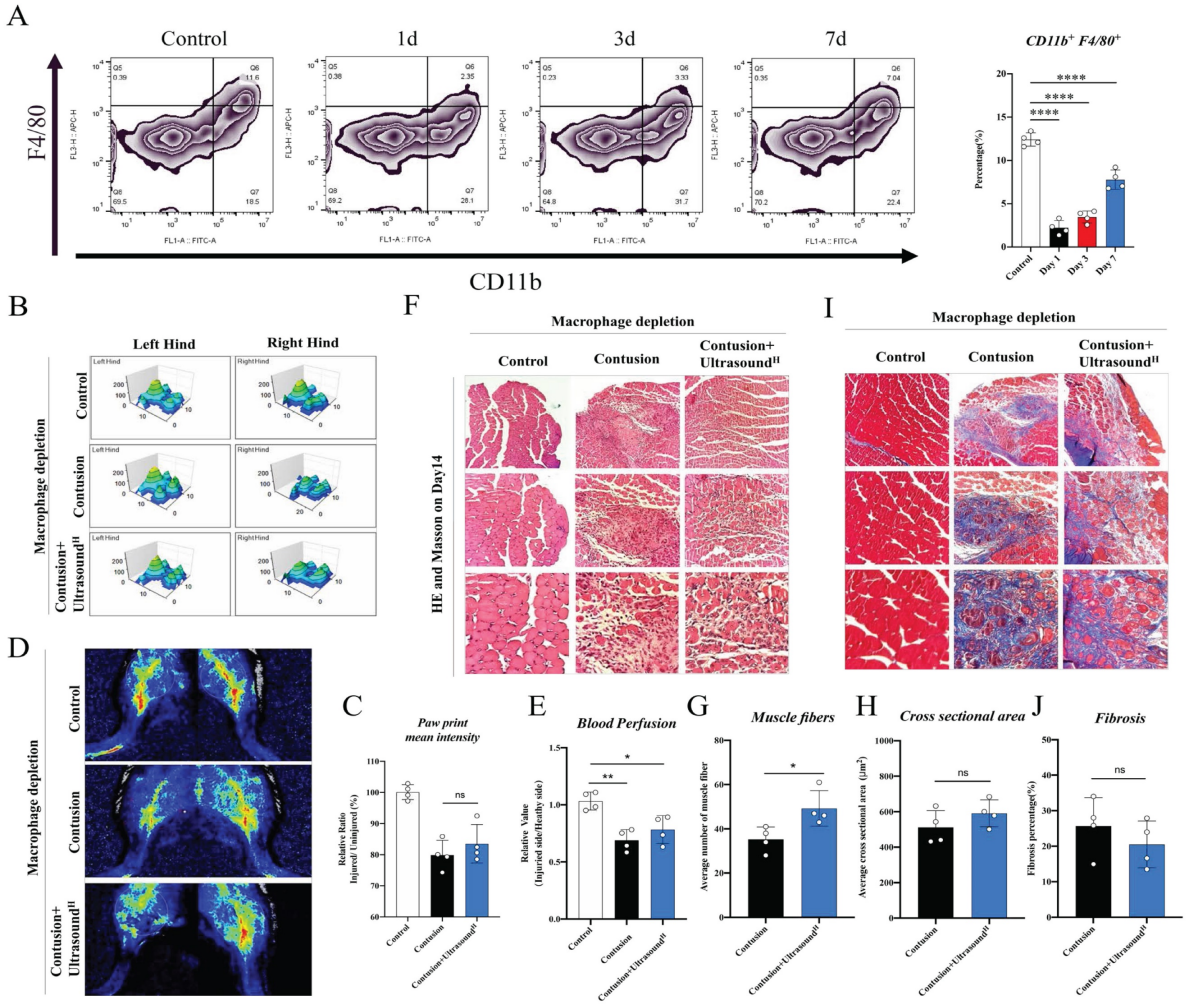
** The therapeutic effect of LIPUS was suppressed in macrophage-depleted mice.** (A) Representative flow cytometry plots showing the percentages of F4/80 and CD11b phenotype in macrophage-depleted mice on Day 1, Day 3, Day 7, and normal mice. The proportion of macrophages in spleen after macrophage depletion was compared with normal mice. (n=4) ****P < 0.0001. (B-C) The 3D footprint intensities images of the uninjured left hind foot and injured right hind foot in macrophage-depleted mice on Day 14. (n=3) No statistically significant improvement was found between the Contusion +Ultrasound^H^ group and the Contusion group. (n=4) ns P>0.05. (D-E) Representative blood perfusion images of the bilateral gastrocnemius muscle in macrophage-depleted mice on Day 14. Circle marked the aimed injured area. The ratio of blood perfusion (injured/uninjured) of lower limbs in different groups was calculated and compared. No statistically significant improvement was found between the Contusion +Ultrasound^H^ group and the Contusion group. (n=4) ns P>0.05. (F-J) Representative HE staining images and Masson staining of injured muscle with macrophages depletion among different groups on Day 14. The scale bar from top to bottom is 250μm, 100μm, and 50μm. Muscle fibers on Day 14 were calculated in different groups. (n=4) *P<0.05. Cross-sectional area and fibrosis percentage on Day 14 among different groups were compared. (n=4) ns P>0.05.

To further confirm the pivotal role of macrophages in LIPUS-mediated skeletal muscle repair, a macrophages depletion model was built. Flow cytometry results of tissue (CD11b+F4/80+ cells were significantly reduced) demonstrated that the content of macrophages in spleen was significantly reduced for the first 3 days with clodronate liposomes injection, and the effects lasted for 7 days long (Fig. 5A). After the effectiveness of the depletion was determined and the contusion model was established, LIPUS was performed for 7 consecutive days. The LIPUS-mediated positive effect on muscle recovery disappeared when analyzed the mice on Day 14. From the behavioral data measured on Day 14, gait and blood perfusion remained unchanged when groups compared with each other (Fig. 5B-E). Besides, the muscle fibers were still as disorganized as the Contusion group, no obvious restoration of CSA and new fibers was observed (Fig. 5F-H). Similarly, the fibrotic area between the Contusion and Contusion+Ultrasound^H^ groups was not considerably different (Fig. 5I-J and Fig. S6). Thus, the macrophages depletion experiments suggested that delaying the macrophage response impairs muscle recovery as shown by Figure 5C, 5E, 5G, 5H, and 5J.

In general, we concluded that the inflammatory microenvironment in injured skeletal muscle can be shifted by LIPUS treatment, and the retreat of inflammation was achieved by the transformation of macrophages from the pro-inflammatory type to the anti-inflammatory type. In general, during the acute inflammatory phase after contusion injury, macrophages are required for LIPUS treatment to beneficially impact muscle regenerative capability by polarizing them toward an anti-inflammatory (M2) phenotype.

### LIPUS promotes M2 polarization via the WNT signaling pathway in vitro

**Fig 6:**
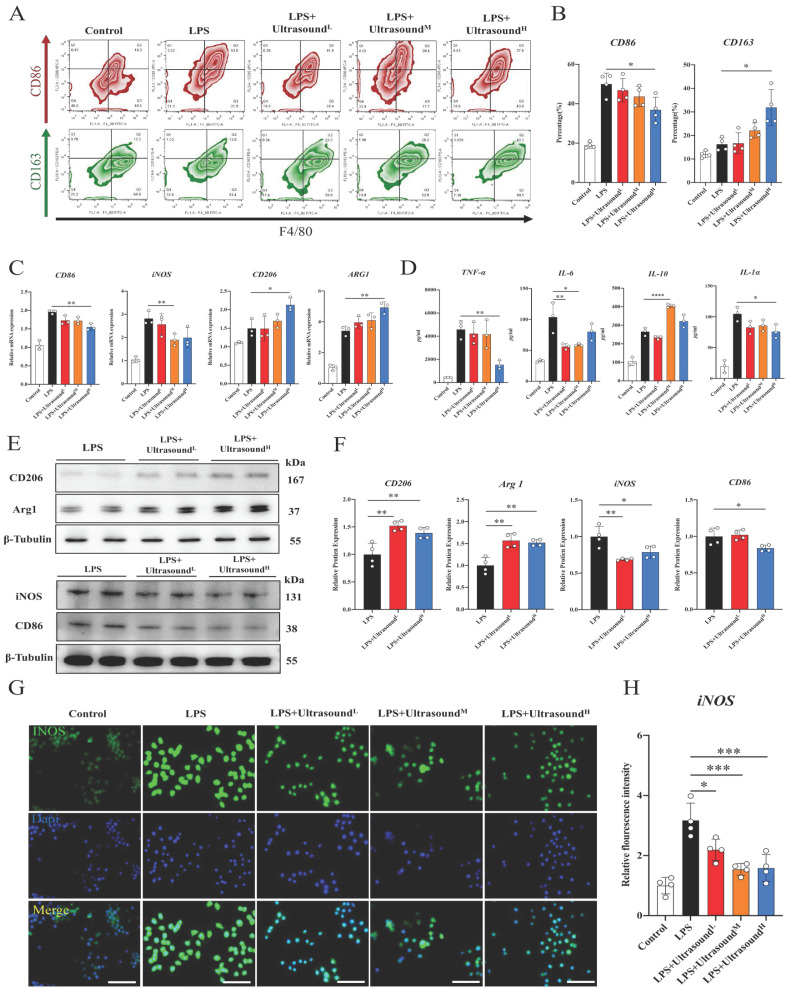
** LIPUS promoted M2 macrophage polarization *in vitro.*** (A) Representative flow cytometry plots showing the percentages of M1 (CD86 + CD4/80+) and M2 (CD163 + CD4/80+) phenotype in macrophages after LIPUS treatment. Quantification of flow cytometry data after two ultrasound stimulation sessions with LPS-induced RAW 264.7 cells. (n=4) *P < 0.05. (C) The gene expression level of CD86, iNOS, ARG1, and CD206 in LPS-induced RAW 264.7 cells were detected by qPCR. (n=3) *P<0.05 **P<0.01. (D) The concentration of cytokine IL-6, IL-1α, TNF-α, and IL-10 in supernatants of LPS-stimulated RAW 264.7 cells after treated were measured by Elisa assay (n=4). *P < 0.05 **P<0.01 ****P < 0.0001. (E-F) The protein expression of CD206, Arg 1, iNOS, and CD86 in LPS-stimulated RAW 264.7 was determined by Western Blot. (n=4) *P<0.05 **P<0.01 ***P<0.001. (G-H) Immunofluorescence intensity changes of iNOS (green), a pro-inflammatory cytokine after treatment with different intensity of ultrasound. The nuclei were dyed with Dapi (blue). Scale=25μm Relative fluorescence intensity (normalized to Dapi) of iNOS was determined. (n=4). *P < 0.05. ***P < 0.001.

To further figure out the connection between specific cellular and molecular components of reduced inflammation by LIPUS, LPS was utilized to induce polarization of macrophages towards M1 in vitro, which could simulate the early inflammatory microenvironment caused by contusion injury in vivo (The operation process of ultrasound for cells is shown in Supplementary Movie 6). Flow cytometry analysis showed that LPS polarized at least ~30% of macrophages toward M1, and the cell morphology directly reflected the induction success (Fig. 6A-B, Fig. S8). Additionally, Increased expression of pro-inflammatory genes, proteins, and cytokines proved LPS-induced activation of macrophage-associated pro-inflammatory pathways at three levels, including iNOS, CD86, TNF-α, IL-1α, and IL-6. The above results verified that LPS-induced inflammation conforms to the early inflammatory microenvironment after skeletal muscle injury. LPS as a classic method simulating the inflammatory environment in vitro has been widely reported in research. Through three different intensifications of standardized intervention, the amount of M1 macrophage presented a downward trend, and the decline was most apparent when a high dose was conducted (about 15%). Meanwhile, the proportion of M2 macrophages was significantly up-regulated after reaching a certain range of stimulation intensity (approximately 20%) (Fig. 6A-B). The PCR, WB, and immunofluorescence analysis also verified these LIPUS-regulated cytological effects. That is, genes and proteins of M2 (CD206 and Arg1) increased, while those of M1 (CD86 and iNOS) decreased (Fig. 6C, E, F). The same is compliant for secreted cytokines (TNF-α, IL-6, IL-1α, and IL-10), with a decrease in the expression and distribution of pro-inflammatory factors (iNOS) (Fig. 6D, G, H). However, it can be observed that the regulation effect of LPS+Ultrasound^L^ group and LPS+Ultrasound^M^ group on LPS-induced inflammatory microenvironment is not ideal compared with LPS+Ultrasound^H^ group.

**Fig 7:**
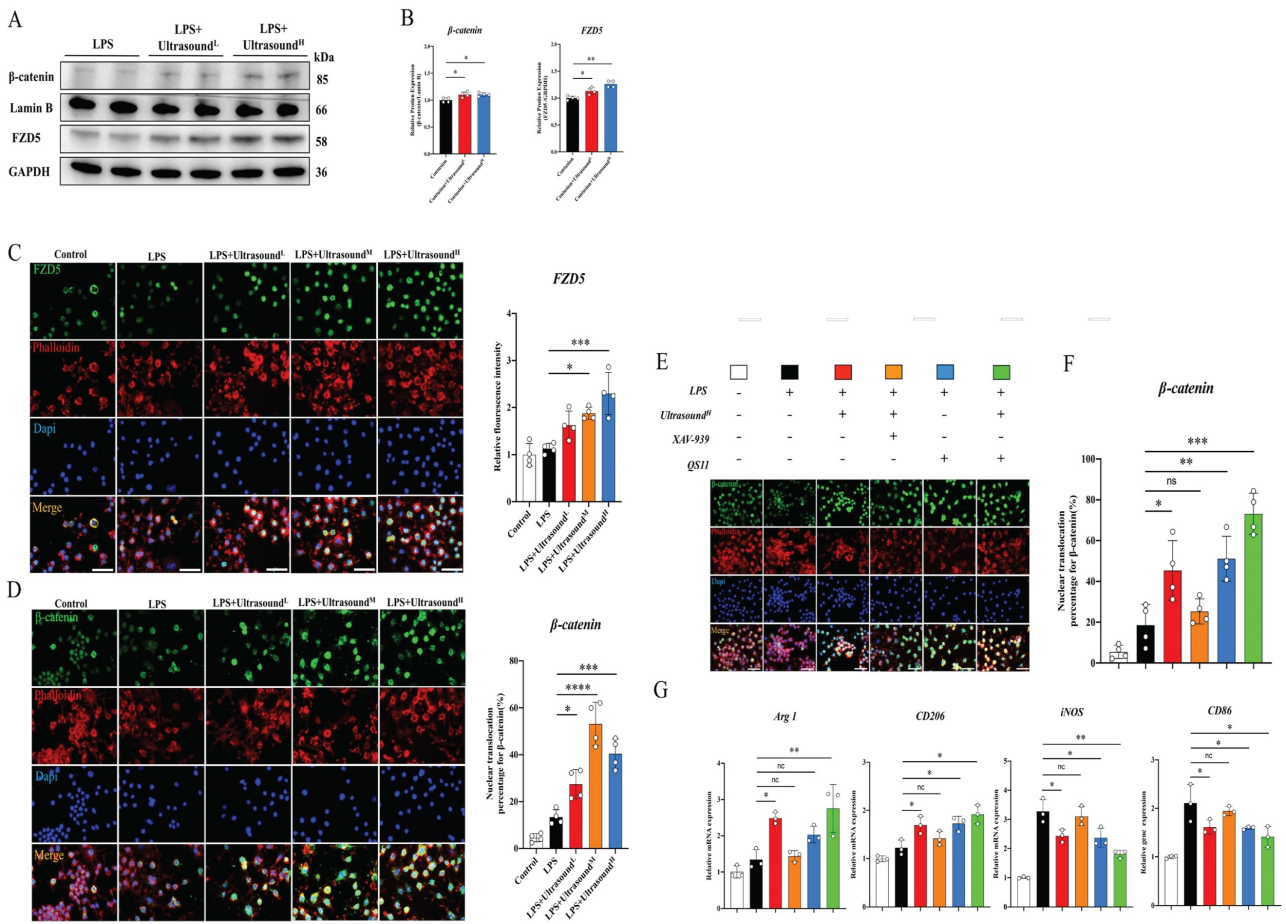
** LIPUS regulated macrophage polarization through the WNT pathway in LPS-induced M1 macrophages.** (A-B) The changes in protein expression of FZD5 and β-catenin in LPS-stimulated RAW 264.7 cells were exposed and recorded over two times of LIPUS treatment. Relative protein expression of β-catenin was calculated after normalized with Lamin B1. (n=4). *P < 0.05. Relative protein expression of FZD5 was calculated after normalized with GAPDH. (n=4). **P < 0.01. (C) Immunofluorescence staining of FZD5(green) expressed on the cell membrane and Phalloidin (red) of macrophage in different groups after treatment with LIPUS. The nuclei were dyed with Dapi (blue). Scale bar=25μm. The relative fluorescence intensity of FZD5 was compared among different groups. (n=4). *P < 0.05. ***P < 0.001. (D)Images showed Immunofluorescence staining of β-catenin(green) expressed in the nucleus, and Phalloidin (red) of macrophages in different groups after treatment with LIPUS. The nuclei were dyed with Dapi (blue). Scale bar=25μm. The nuclear translocation percentage for β-catenin was compared among different groups. (n=4) *P < 0.05 ***P < 0.001 ****P<0.0001. (E-F) Immunofluorescence staining of β-catenin(green) expressed in the nucleus, and Phalloidin (red) of macrophage in different groups after treating different combinations of UltrasoundH, XAV-939, and QS11. The nuclei were dyed with Dapi (blue). Scale bar=25μm. The nuclear translocation percentage for β-catenin was compared among different groups. (n=4) *P < 0.05 **P < 0.01 ***P<0.001. No significant changes were found when LIPUS and XAV-939 were combined. (G) The gene expression level of CD86, iNOS, ARG1, and CD206 in different groups after treating different combinations of LIPUS, ultrasoundH, XAV-939, and QS11 were detected by qPCR. (n=3)

The potential molecular mechanism of macrophages alteration raised by LIPUS is still unclear. After exploration and screening, we found that the WNT pathway, a classical pathway regulating the polarization of macrophages, figured prominently during this process. Before exploring the effect of LIPUS on this pathway, it is reported that both FZD1 and FZD5 receptors may be involved in the process of macrophage polarization in Frizzled (FZD) family. After analyzing the effect of LIPUS on the expression of both receptors *in vitro,* we found that FZD5 was significantly increased, while the expression of FZD1 was not significantly altered (Fig. S9). Then, we investigated the expression of FZD5 and nuclear β-catenin through WB, which are the receptor and downstream mediator of WNT signaling respectively. In the treatment groups, both proteins were significantly up-regulated (Fig. 7A-B). From the immunofluorescence analysis, with the expression of FZD5 increased after intervention, the number of β-catenin nuclear entries was spatially increased, histologically proving that LIPUS activated the WNT pathway (Fig. 7C-D). The conclusion can be established through the above results that LIPUS promoted M2 polarization but reduced M1 polarization via activating the WNT signaling pathway, subsequently reducing the secretion of inflammatory factors, and modulating the whole inflammatory microenvironment.

In order to ascertain the participation of the WNT pathway in LIPUS-mediated M2 polarization, the respective combination of LIPUS with XAV-939 (WNT/β-Catenin signaling inhibitors) 53 and LIPUS with QS11 (WNT/β-Catenin signaling agonists) 46 was used to treat the LPS-induced macrophages. The results showed that β-catenin entering the nucleus and the therapeutic effect of ultrasound on RAW 264.7 were significantly impeded when the XAV-939 existed (Fig. 7E-F). A synergistic increase was observed when both QS11 and LIPUS were involved. The result of the WNT pathway-related gene analysis (Arg1, iNOS, CD206, and CD86) was consistent with immunofluorescence, which was that XAV-939 blocked the LIPUS-medicated effect while QS11 promoted the effect (Fig. 7G).

### LIPUS promotes M2 polarization via WNT signaling pathway in vivo

**Fig 8:**
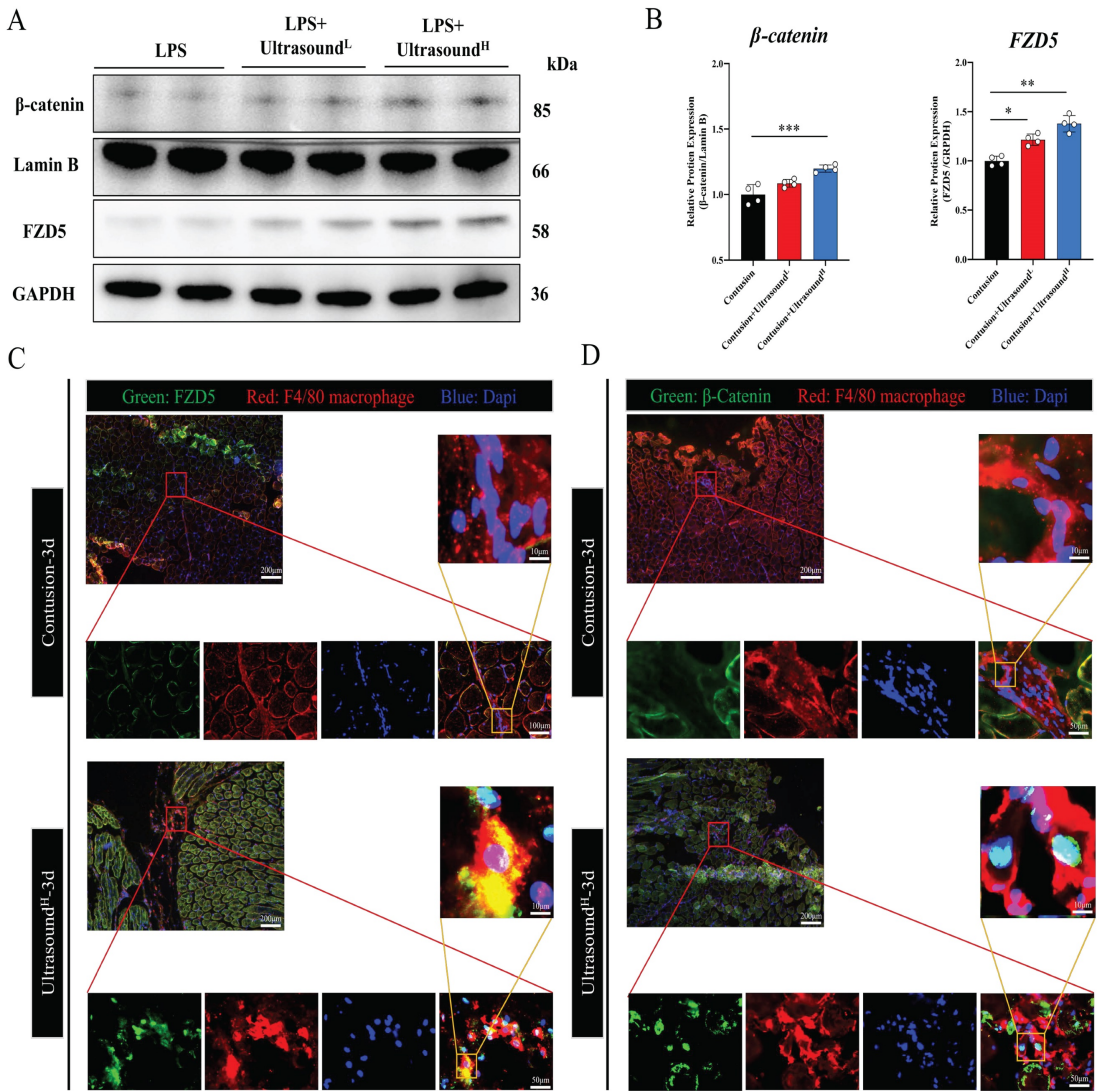
** LIPUS promoted M2 macrophage polarization through the Wnt pathway *in vivo.*** (A-B) The protein expressions of FZD5 and β-catenin after being treated with LIPUS in contusion-injured mice were determined by Western Blot. (n=4) *P<0.05 **P<0.01 ***P<0.001. (C-D) Immunofluorescence was used to detect the relative expression and distribution of FZD5/β-catenin on Day 3 after acute injury with or without LIPUS treatment. Scale bar=200, 50, or 25μm.

In order to confirm whether the WNT pathway also activated after LIPUS treatment in vivo, we further detected the protein expression of FZD5 and β-catenin in LIPUS-treated mice. The WB results from mice on Day 3 with LIPUS treatment showed an increase in expression of FZD5 and nucleated β-catenin, especially in the Contusion+ultrasound^H^ group (Fig. 8A-B). In addition, immunofluorescence was used to observe the spatial expression of FZD5 and nucleated β-catenin in macrophages on Day 3 and the images showed that the WNT signaling pathway in macrophages was indeed activated after high-dose LIPUS treatment, which was consistent with the results of WB and above cell experiments (Fig. 8C-D).

**Fig 9:**
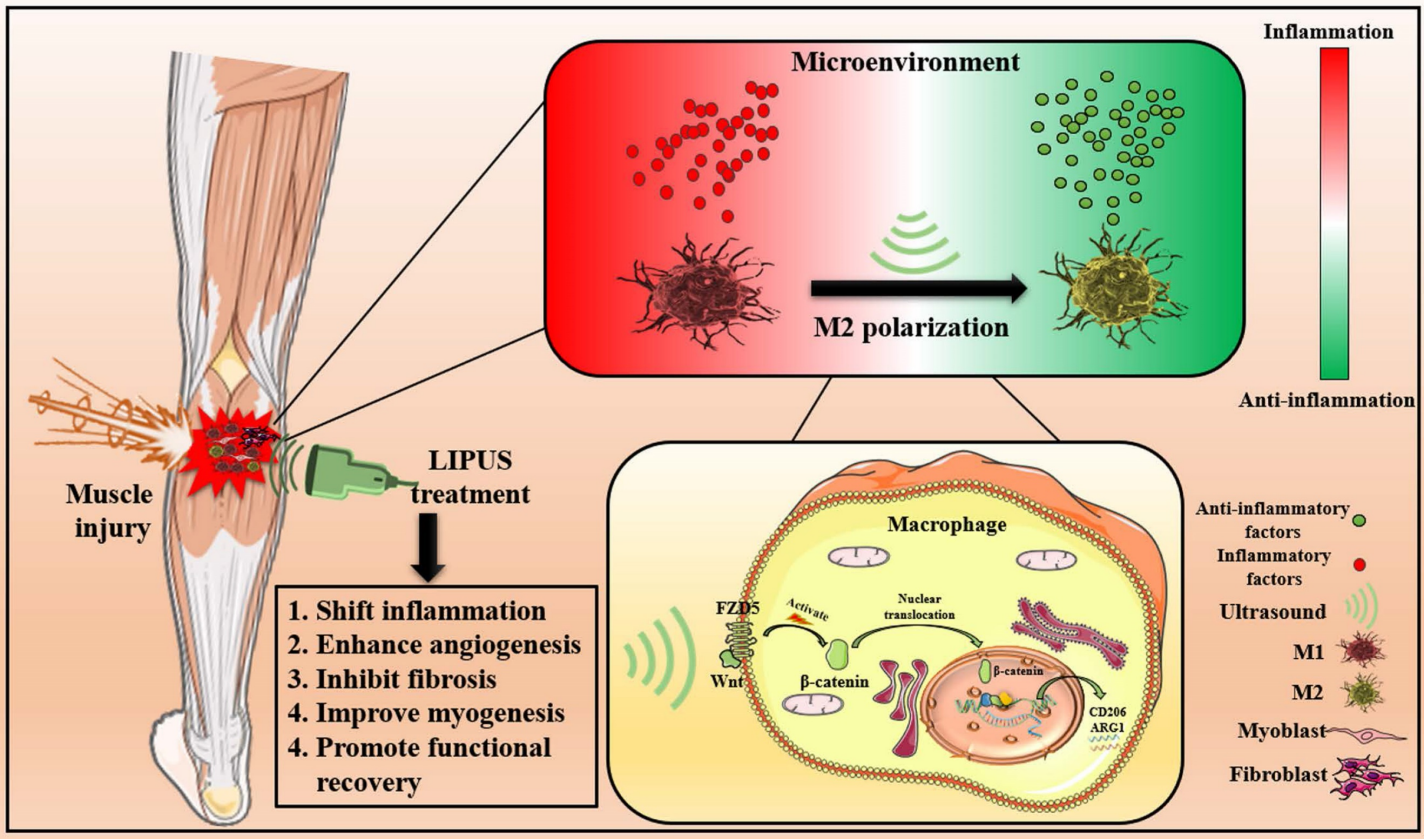
** Schematic depiction of this work.** The pathophysiological mechanism of LIPUS treatment of muscle injury mainly includes relieving inflammation, enhancing angiogenesis, inhibiting fibrosis, improving myogenesis, and promoting functional recovery. At the level of molecular mechanism, LIPUS regulates macrophage polarization by activating the WNT pathway and finally reversing the hyperinflammatory microenvironment.

The results above indicated that LIPUS modulated M1 and M2 macrophages polarization by activating WNT signaling pathway, subsequently changing the expression of inflammation-related mRNA, proteins, and cytokines from *in vitro.*, respectively. These molecular changes ultimately promoted the reversion of the inflammatory microenvironment. Excessive inflammatory microenvironment remission eventually results in angiogenesis, inhibits fibrosis, improves myogenesis, and promotes functional recovery (Fig. 9).

## Discussion

In this study, it has been thoroughly revealed that LIPUS treatment could promote muscle healing elicited by contusion via modulating the inflammatory immune microenvironment. The multiple results of all-inclusive functional tests indirectly proved the functional recovery of the injured muscle after LIPUS treatment. Meanwhile, Muscle mechanical testing directly proved therapeutic effects of LIPUS. According to *in vivo* and *in vitro* explorations, we proposed that the beneficial effects of LIPUS treatment relied on the regulation of macrophage polarization via mechanical activation of WNT signaling.

The spatio-temporal balance between M1 and M2 macrophages is of great significance for orchestrating the inflammatory immune microenvironment after severe muscle injury 51,54. Disturbing macrophages polarization not only extends inflammation but also leads to the formation of fibrosis, which has considerable impacts on skeletal muscle function and can increase the risk of secondary injury 54-56. Therefore, an adjuvant treatment necessary is required to reach an appropriate muscle healing. The therapeutic ultrasound technique was first proposed by Corradi et al. 57 and its effectivity in tissues repairs e.g. bone fractures, stroke, chronic prostatitis, tendon healing, ligament healing, inter-vertebral disc resorption, and cartilage recovery has been proved 58,59. Although LIPUS was first used by David Lindsay in skeletal muscle injuries in 1990 for athletic muscle injury 60, its biological mechanisms have not yet been fully elucidated, with known biomechanical mechanism such as stimulation of mechanically sensitive membrane surface receptors 61,62, modulation of conformation state of ion channels 61,63, and changes of membrane capacitance 64. To date, many studies have attempted to elucidate the underlying mechanisms involved in the therapeutic effects of LIPUS on skeletal muscle injury in basic animal models. For example, Chongsatientam and Yimlamai found that LIPUS could hastened muscle recovery by upregulating angiogenesis in a rat model of gastrocnemius contusion injury 65. Negata et al. discovered that LIPUS modulated the inflammatory response, increased activated satellite cells expressing Pax7, and up-regulated the myogenic regulatory factor in a mice model of Cardiotoxin-induced muscle injury 66. Silveira et al. detected that LIPUS can alleviated the oxidative stress to improve muscle healing in a rat model of gastrocnemius contusion injury 67. Without exception they failed to assess the functional recovery of skeletal muscle-related indicators comprehensively enough, nor had they explored the mechanisms in depth.

In recent studies regarding the therapeutic mechanism of LIPUS in other clinical models, deeper biological mechanisms of LIPUS have been explored. Li et al proved that ultrasound could control anti-inflammatory polarization of microglia for targeted ischemic stroke therapy. Meanwhile, ultrasound combined with microglial therapy may be a novel strategy for stroke treatment via creating anti-inflammatory microenvironment 68. Based on Zhang et al., 69 LIPUS promoted spinal fusion and stimulated the transition of M1 to M2 macrophages. In the heart system, Zhao et al. demonstrated that LIPUS prevents hypoxia-induced cardiac fibrosis through HIF-1α/DNMT3a pathway via a TRAAK-dependent manner 61. Their team also discovered that LIPUS could ameliorate angiotensin II-induced cardiac fibrosis by alleviating inflammation via a caveolin-1-dependent pathway 62. In our study, various histological and behavioral functional indicators have been evaluated in detail and specific mechanisms have been elucidated by which LIPUS regulates the immune microenvironment of skeletal muscle from an immunological perspective.

As for LIPUS parameters set in vivo, human's exposure area and energy are 30mW/cm^2^ for human mouthpieces 70, whereas Montalti et al. 19 reported that rats are 30mW/cm^2^ for tibialis anterior. In addition, Chan's settings are 30mW/cm^2^ for gastrocnemius muscles 17. Because some studies are inconsistent 71,72, we performed pre-experiments and it was determined that 30 mW/cm^2^ and 60mW/cm^2^ are the lowest and highest energy to promote proper muscle healing, which is equivalent to 1 or 2 times the energy used in humans 70. For the cell experiments, Zhao et al. 73 applied the settings of 200 mW/cm^2^ and 1.5 MHz on macrophages, while other researchers 74,75 applied 50 mW/cm^2^ on PC12 cell and 30 mW/cm^2^ on C2C12 cells. Furthermore, it was determined that the dose range macrophages can tolerate is from 10 to 90 mW/cm^2^ through pre-experiments. The above parameters provide strong support for future studies on LIPUS treatment on skeletal muscle or macrophages.

Our study proved that ultrasound reduced early cell infiltration and hematomas histologically, which is in accordance to the previous results conducted by Signori and Junior et al. 39,76. In recent study by Sabbagh et al., they reported that LIPUS could opened the brain blood barrier and modulate liberation of chemokine, and the rapid exit of inflammation and hematoma in our study may be elicited by LIPUS-mediated vascular permeability and chemokines changes 77. In addition, new muscle fibers number and CSA elevated after LIPUS treatment in our study suggested that LIPUS can promote muscle healing and recover muscle strength. Stress is a more accurate representation of the muscle specific mechanical (material) properties. Fibrosis was significantly reduced in the LIPUS group, which also demonstrated that skeletal muscle achieved suitable tissue remodeling. Those results were also similar to the previous study 9. Moreover, LASCA is now one of the gold standards for assessing vascular status 78 and the LASCA was innovatively applied to assess vascular regeneration in LIPUS treatment of muscle injury for the first time, which further demonstrated that muscle healing is significantly enhanced by LIPUS. Wang et al. 79 found a significant recovery of blood flow after LIPUS treatment in the abusive head trauma model, which is consistent with our results. What's more, behaviorally and functionally, performed gait analysis, force test, rotard rod test, treadmills test have been comprehensively performed on injured mice and it was found that LIPUS treatment does significantly improv muscle function, including a range of indicators such as force, physical co-ordination, persistence, and posture, which is consistent with the recovery force reported by Yimlamai and Chan et al. 17,80, further providing a solid basis for the application of LIPUS in clinical practice.

Macrophage polarization has received extensive attention in many animal models of injurious disease 27,81-84. For instance, Martins et al. 51 found that increasing the M2 subtype at the early stage reduced fibrosis formation in the muscle contusion model, which was consistent with our results that LIPUS reduced fibrosis formation through mediating macrophage polarization. Zhou et al. 85 demonstrated M2 derived exosomal miR-501 could promote myotube formation after muscle injury. These studies proved the importance of macrophage polarization regulation in the process of tissue repair 27,86,87. Based on our previous studies, BMSC-derived exosomes can promote M2 polarization in skeletal muscle, which remarkably improved skeletal muscle healing 31, and inflammatory C2C12-Exos can induce M1 polarization to prolong the inflammatory response 28. In the present study, it was found that early overall macrophage infiltration was reduced, and it appeared that macrophage was a negative factor. But after we knocked out macrophages using clodronate liposomes, we found that LIPUS-mediated promotion of skeletal muscle healing was greatly reduced, both histologically and behaviorally, and the LIPUS+contusion groups showed no significant differences compared with contusion-only group, which suggested that macrophages played an indispensable role in LIPUS-mediated therapeutic effects and that the absence of M2 macrophages would lead to dysregulated tissue remodeling.^27^,^88^ This similar finding was also demonstrated in the study of Xiao et al. After macrophage depletion, there were significantly more fibrosis areas in the injured muscle 89.

Afterward, how LIPUS regulates macrophages was explored, and it was identified that LIPUS promoted M2 and reduced M1macrophages based on both *in vitro* and *in vivo* models, which was in line with the findings of Junior's study 39. We further clarified the related underlying mechanisms in the cell model and demonstrated that the WNT/FZD5/β-catenin axis was the key pathway in regulating LIPUS promoting M2 polarization through positive and negative validation *in vitro*. WNT/β-catenin is an evolutionarily highly conserved fundamental signaling system and orchestra the proliferation, differentiation, apoptosis, motility, and polarization of cells through the WNT ligands binding to FZD receptors and β-catenin nuclear translocation 90. 19 WNT homologs and 10 receptors of the FZD family as well as several co- and alternative receptors are conserved in mice and men. After a certain literature research, it was discovered that WNT3a and WNT5a can regulate the polarization of macrophages via FZD1 and FZD5 receptors respectively 91-93. However, with ultrasonic stimulation, we found that FZD1 protein expression was not significantly altered whereas FZD5 protein expression was dramatically elevated *in vitro*. Therefore, it can be concluded that FZD5 was involved in modulating macrophage polarization.

Although there is still no research directly demonstrating that FZD family receptors belong to mechanosensitive membrane surface receptors, some articles indicated the connection between FZD and other mechanosensitive receptors, such as integrin and caveolin 94. Whether the mechanical energy generated by ultrasound acts directly on FZD or modulates FZD through other known mechanosensitive ion channels or membrane surface receptors still requires further investigation.

Most importantly, this mechanism was evaluated* in vivo*, which was consistent with *in vitro* experiments. The WNT signaling pathway was reported as a classical pathway that regulates macrophage polarization 95-98. In term of the study by Cosin-Roger et al. 99, it was found that the promotion of the WNT pathway could enhance M2 polarization, which results in the promotion of mucosal repair in TNBS-Treated mice. However, LIPUS has been found to inhibit the WNT pathway in the synoviocyte in Liao's study 100. Nevertheless, in Ren's study 16, it was identified to promote the WNT pathway. Besides, Li et al. demonstrated that LIPUS could promote the differentiation of HGF-induced BMSCs into hepatocytes through the Wnt/β-catenin signaling pathway 101. Therefore, the effects of LIPUS on WNT signaling are different depending on the tissue and cell types 16,100. Since the WNT pathway plays a role in muscle, it is also worthwhile to investigated weather LIPUS promote the muscle regeneration through mediating WNT pathway. In this study, we found that ultrasound promoted M2 polarization via WNT pathway. When there were more M2 polarized macrophages, range of anti-inflammatory factors which relieved the excessively inflammatory microenvironment will be produced. The mannered inflammatory microenvironment further benefited tissue remodeling, vascular regeneration and other responses 27,102,103, which also exhibited from histological and behavioral aspects. In conclusion, our research has found that LIPUS promoted muscle healing and functional recovery by promoting M2 polarization through the WNT pathway and alleviating the inflammatory microenvironment.

Although we tried to elucidate the effects of LIPUS on skeletal muscle healing from various perspectives, we still have some shortcomings. First, we did not construct macrophage-specific FZD5 knockout mice to further validate the experimental results. Second, we cannot directly predict the optimal dose of ultrasound for clinical use in humans from this article, and more clinical cohort studies are required to investigate 104. Third, our *in vitro* cell experiments do not fully reflect *in vivo* conditions. Although some studies have reported that LIPUS can promote myoblast growth* in vitro* 17, it is not possible to fully model all cell types within skeletal muscle. The inflammatory microenvironment model induced by LPS is classically and widely applied to investigate macrophage-mediated immune regulation 105,106.

## Conclusion

In this study, we demonstrated for the first time that application of LIPUS in acute stage after muscle injury, especially high-dose strategy (60mW/cm^2^), suppressed the inflammatory immune microenvironment and improved muscle healing, where promoting M2 polarization via regulation of WNT signaling pathways was the key mechanism involved. Besides, the multiple results of comprehensive functional tests indirectly proved the functional recovery of the injured muscle after LIPUS treatment. Our work presents new insight and convincing evidence for LIPUS-based noninvasive treatments of severe muscle injury and other acute inflammatory diseases.

## Supplementary Material

Supplementary figures, data, and movie legends.

Supplementary movie 1.

Supplementary movie 2.

Supplementary movie 3.

Supplementary movie 4.

Supplementary movie 5.

Supplementary movie 6.

## Figures and Tables

**Table 1:** Primers for PCR used in the experiment

Gene name	Forward primer	Reverse primer
CD86	TGACCGTTGTGTGTGTTCTGGA	TCTCTGTCAGCGTTACTATCCCG
CD206	GCTGGCGAGCATCAAGAGTA	AGGAAACGGGAGAACCATCAC
Arg1	CATATCTGCCAAAGACATCGTG	GACATCAAAGCTCAGGTGAATC
iNOS	GGGCTGTCACGGAGATCAATG	GCCCGGTACTCATTCTGCATG
GAPDH	CCTCGTCCCGTAGACAAAATG	TGAGGTCAATGAAGGGGTCGT

**Table 2:** Primary antibodies used in the experiment

Antibody	Source	Catalog No.	Type	Dilution	M.W. (kD)
iNOS	Affinity	AF0199	Rabbit mAb	1:1000(W.B.)	130
iNOS	Abcam	ab178945	Rabbit mAb	1:200(IF)	
Arg1	Affinity	DF6657	Rabbit mAb	1:1000(W.B.)	35
CD86	Abcam	ab220188	Rabbit mAb	1:1000(W.B.)	38
				1:200(IF)	
CD206	Abcam	ab64693	Rabbit mAb	1:2000(W.B.)	167
				1:200(IF)	
CD68	Abcam	ab955	Mouse mAb	1:200(IF)	
CD86 PE	eBioscience	12-0862-81	Rat mAb	0.125 μg/test	(Flow cyt)
CD163APC	eBioscience	17-1631-80	Rat mAb	0.25μg/test	(Flow cyt)
F4/80 APC	eBioscience	47-4801-80	Rat mAb	0.125μg/test	(Flow cyt)
F4/80 FITC	eBioscience	11-4801-82	Rat mAb	0.125μg/test	(Flow cyt)
F4/80	Abcam	ab90247	Rat mAb	1:500(IF)	
CD11b FITC	Abcam	ab24874	Rat mAb	0.125 µg/test	(Flow cyt)
β-Catenin	Abcam	ab32572	Rabbit mAb	1:1000(W.B.)	95
				1:100(IF)	
FZD5	Abcam	ab75234	Rabbit mAb	1:1000(W.B.)	65
				1:500(IF)	
Phalloidin	Abcam	ab176757	Mouse mAb	1:1000(IF)	
β-Tubulin	Abcam	ab52623	Rabbit mAb	1:5000(W.B.)	52
Lamin B1	Abcam	ab16048	Rabbit PAb	1:2000(W.B.)	66
GAPDH	Abcam	ab8245	Mouse mAb	1:5000(W.B.)	36
					

## Abbreviations

Low intensity pulsed ultrasound: LIPUS
Myogenic progenitor cells: MPC
Extracellular matrix: ECM
Nitric oxide: NO
Dulbecco's modified eagle medium: DMEM
Fetal bovine serum: FBS
Lipopolysaccharides: LPS
Hematoxylin and eosin: HE
Laser Speckle Contrast Analysis: LASCA
Cell counting kit-8: CCK-8
Creatine kinase: CK
Cross-Sectional areas: CSA
Enzyme-linked immunosorbent assay: ELISA
Room temperature: RT
