# Supplementary material for: Low-intensity pulsed ultrasound promotes skeletal muscle regeneration via modulating the inflammatory immune microenvironment
Source: Int J Biol Sci. 2023 Feb 5;19(4):1123–45. doi: 10.7150/ijbs.79685 (PMC10008697; doi:10.7150/ijbs.79685)
Supplement: Supplementary file 1 — Supplementary figures, data, and movie legends. [file ijbsv19p1123s1.pdf]

Supplemental Figures

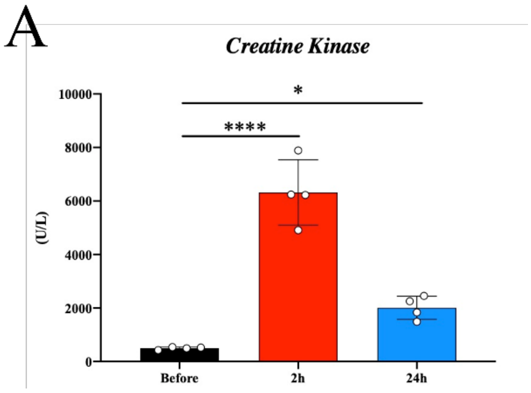

S1. Creatine Kinase concentration changes in contusion-modeled muscle. Related to figure 2.

(A) The serum creatine kinase was analysis at before injured and 2h, 24h after injured. (n=4) *P < 0.05 ****P < 0.0001.

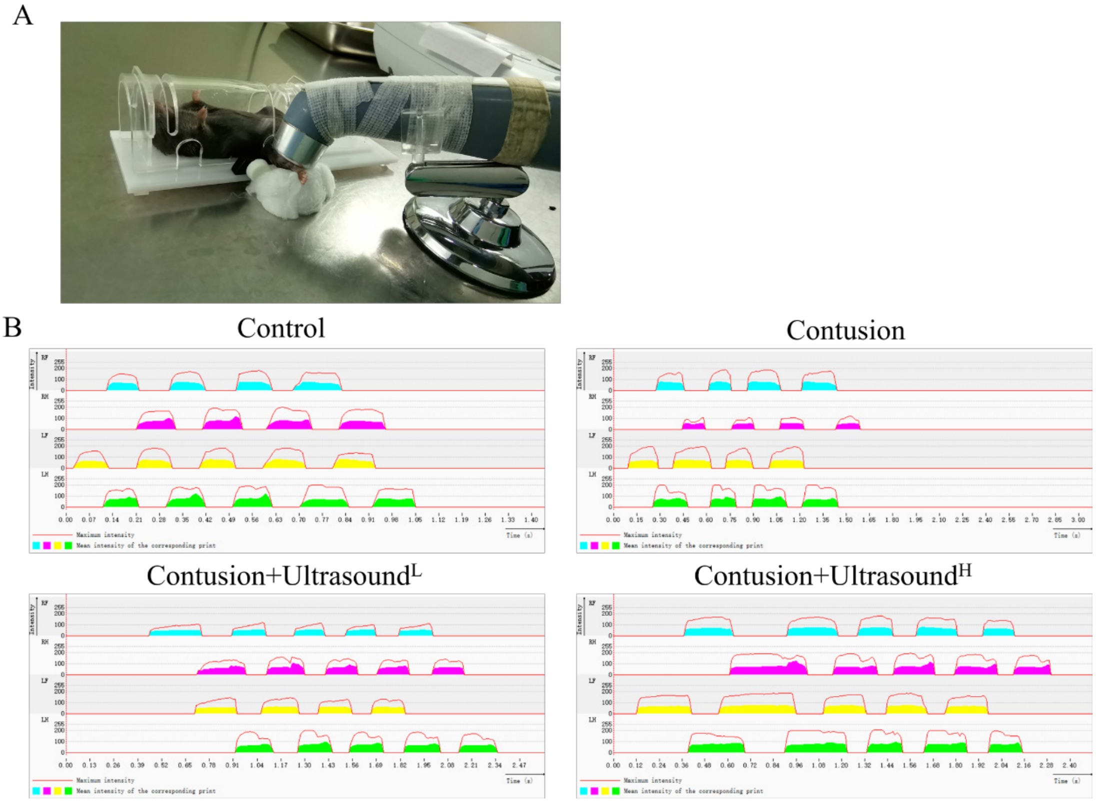

S2. Image of LIPUS treatment in mice and 2D gait patterns. Related to figure 2.

(A) The contusion-modeled mouse was placed in a container to fully expose the right contusion gastrocnemius muscle. LIPUS is then fixed with a designated scaffold to keep the ultrasound transducer parallel to the muscle, and the appropriate amount of coupling agent was applied before treatment.

(B) Representative images of 2D gait patterns of different groups. The difference in gait cycle was visualized.

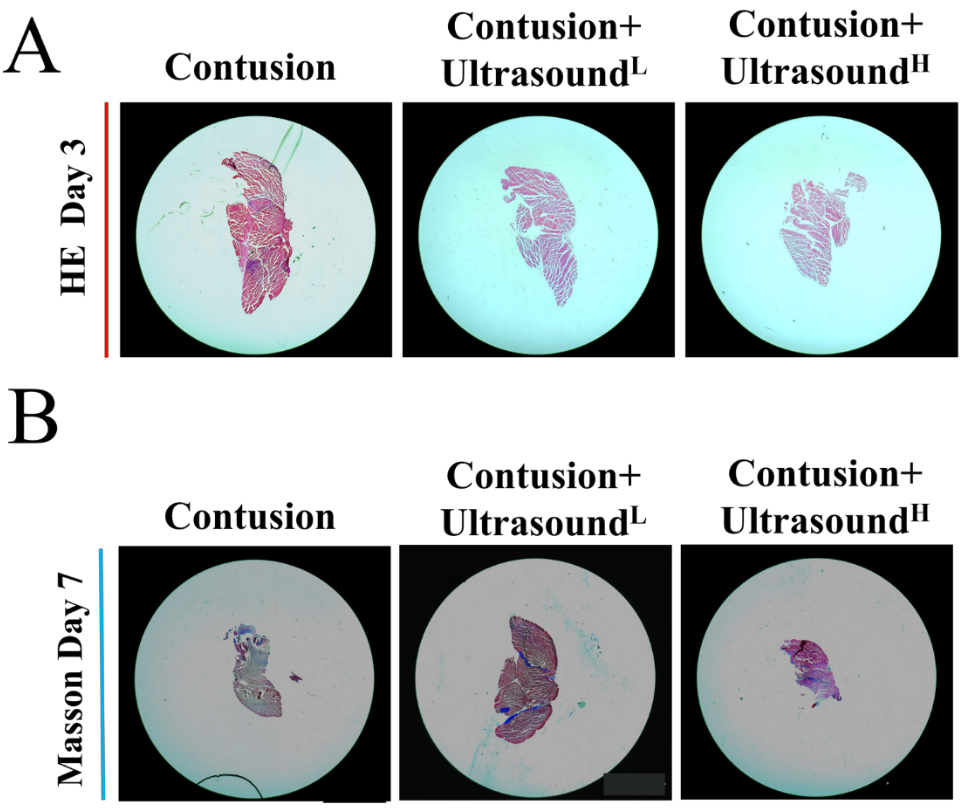

S3. Histological staining images of injured muscle. Related to figure 3.

(A) Representative HE staining images of injured muscle among different groups on Day 3.

(B) Representative Masson staining images of injured muscle among different groups on Day 7.

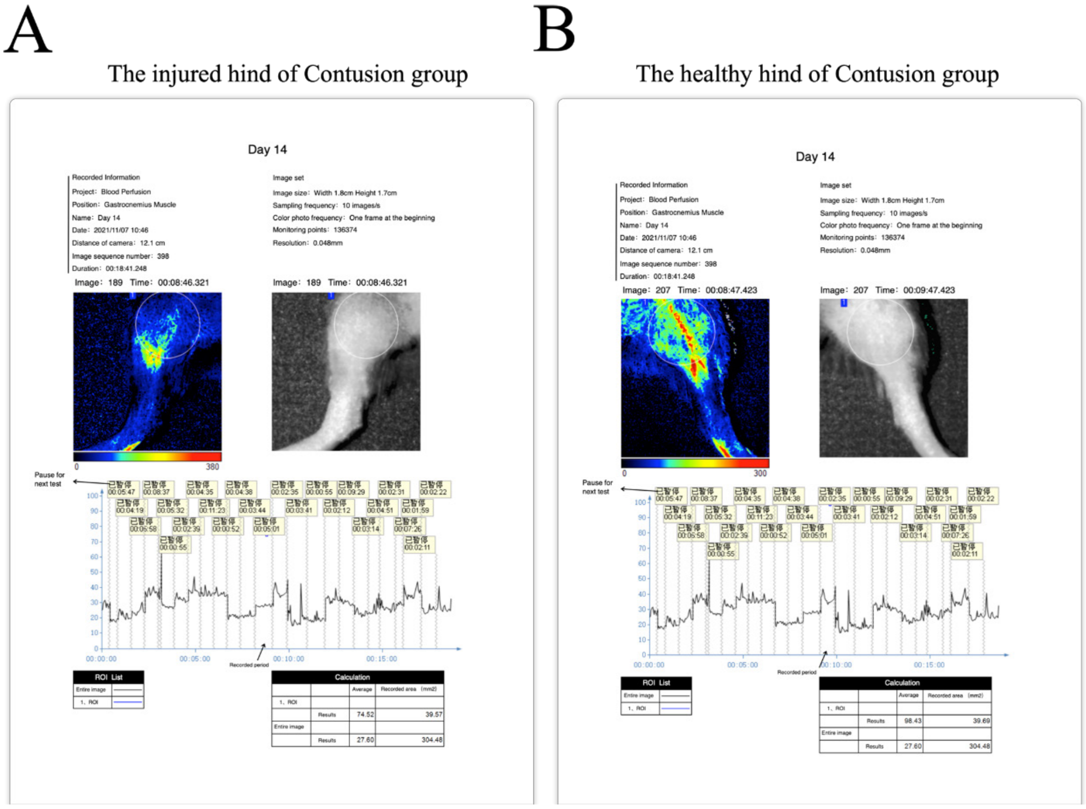

S4. The representative data of blood perfusion in the Contusion group. (A)The injured hind of the Contusion group. (B) The healthy hind of the Contusion group. Related to figure 3.

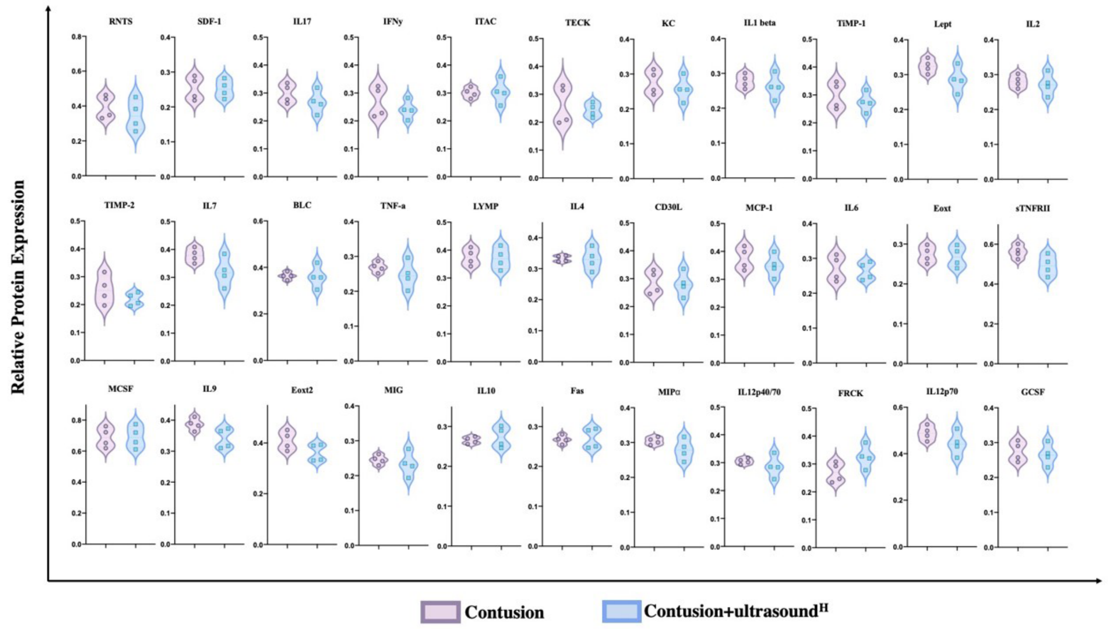

S5. Relative protein expressions of insignificantly changed inflammatory cytokines after LIUPS treatment. Related to figure 4.

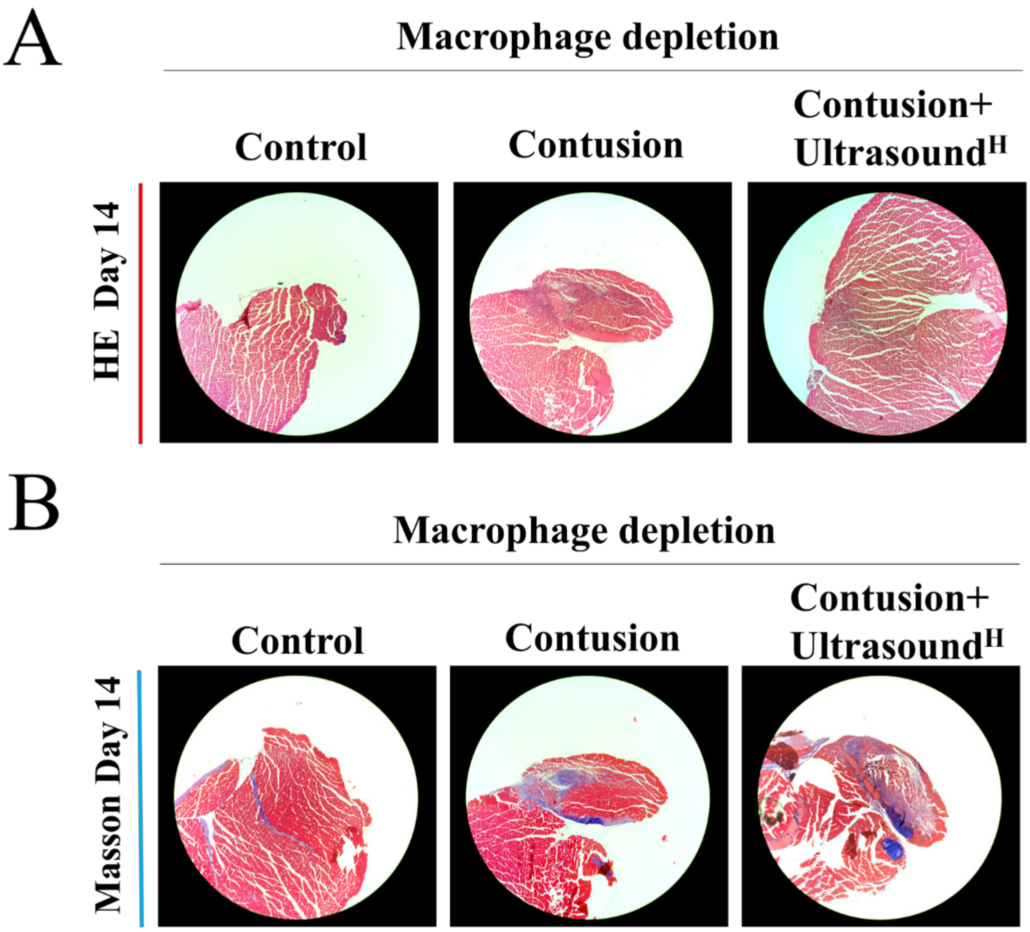

S6. Histological staining images of injured muscle with macrophage depletion. Related to figure 5.

(A) Representative HE staining images of injured muscle with macrophage depletion among different groups on Day 7.

(B) Representative Masson staining images of injured muscle with macrophage depletion among different groups on Day 14.

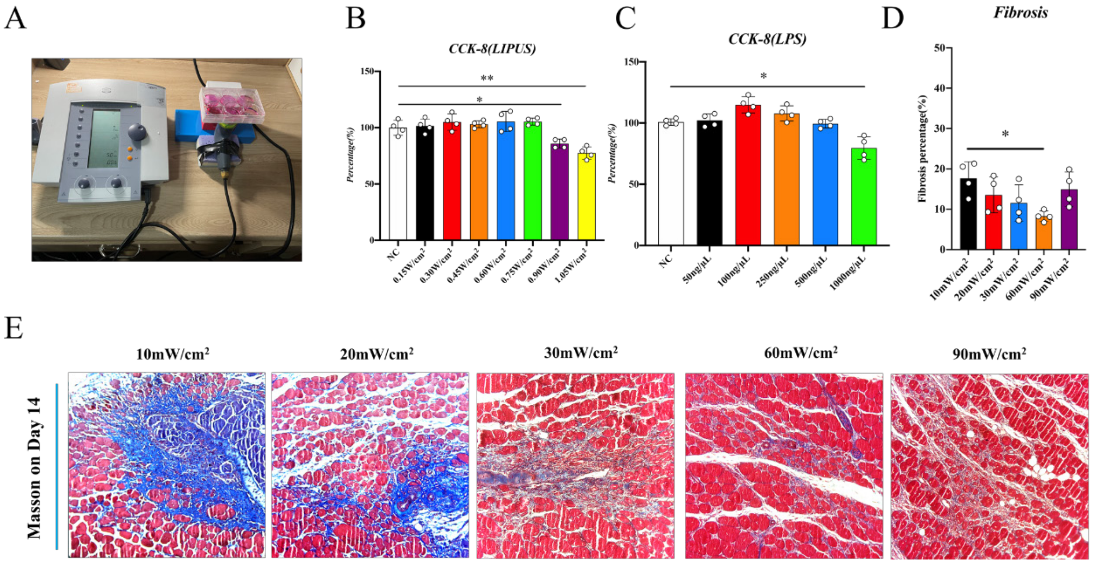

S7. Image of LIPUS treatment on cells and the cellular viability at different LIPUS intensities treatment. Related to figure 6.

(A) We fixed LIPUS on a flat table with the ultrasound treatment head up and placed six-well plates on it to keep them stable and parallel between them. The cells were then treated.

(B) Cell Counting Kit-8 (CCK-8) is tested at different LIPUS intensities treatment for determining range of treatment intensity (n=4) $*P < 0.05$. $**P < 0.01$.

(C) Cell Counting Kit-8 (CCK-8) is tested at different LPS concentration stimulating RAW 264.7 (n=4) $*P < 0.05$.

(D-E) Masson staining is conducted for muscle fibrosis for different LIPUS intensities (10mW/cm^2, 20mW/cm^2,30mW/cm^2, 60mW/cm^2, and 90mW/cm^2) treatment for determining range of treatment intensity (n=4) $*P < 0.05$. The scale bar from top to bottom is 250μm, 100μm, and 50μm. Muscle fibers on Day 14 were calculated in different groups

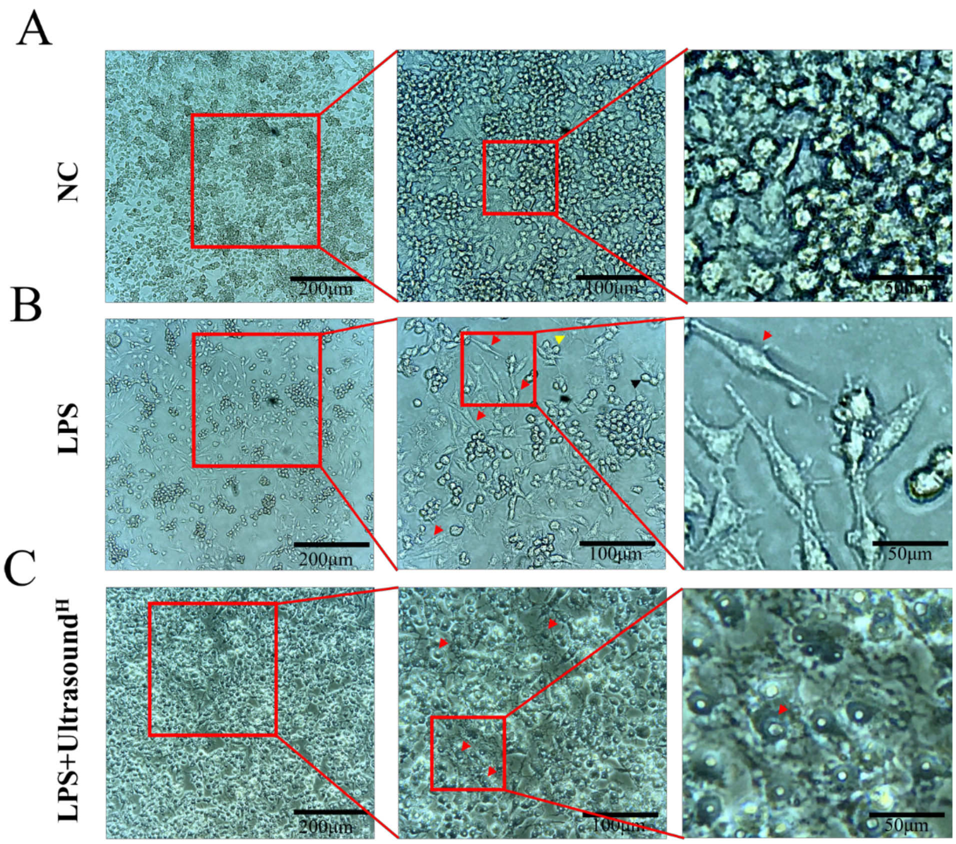

S8. Morphology of LPS-induced M1 macrophages. Related to figure 6.

(A) Negative controlled RAW264.7 cell morphology under microscope. Scale bar=200, 100, or 50μm.

(B) LPS-induced RAW264.7 cell morphology under microscope. Scale bar=200, 100, or 50μm.

(C) LIPUS-treated and LPS-induced RAW264.7 cell morphology under microscope. Scale bar=200, 100, or 50μm.

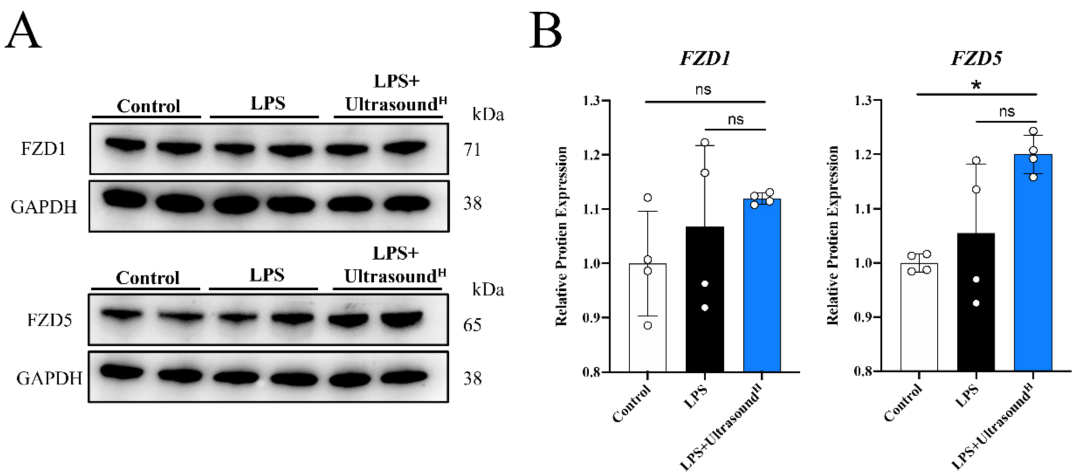

S9. LIPUS regulated the expression of FZD5 *in vitro*. Related to figure 7.

(A-B) Relative protein expression of FZD5 and FZD1 in RAW 264.7 after three different condition treatment was calculated after normalized with GAPDH. (n=4). *P < 0.05.

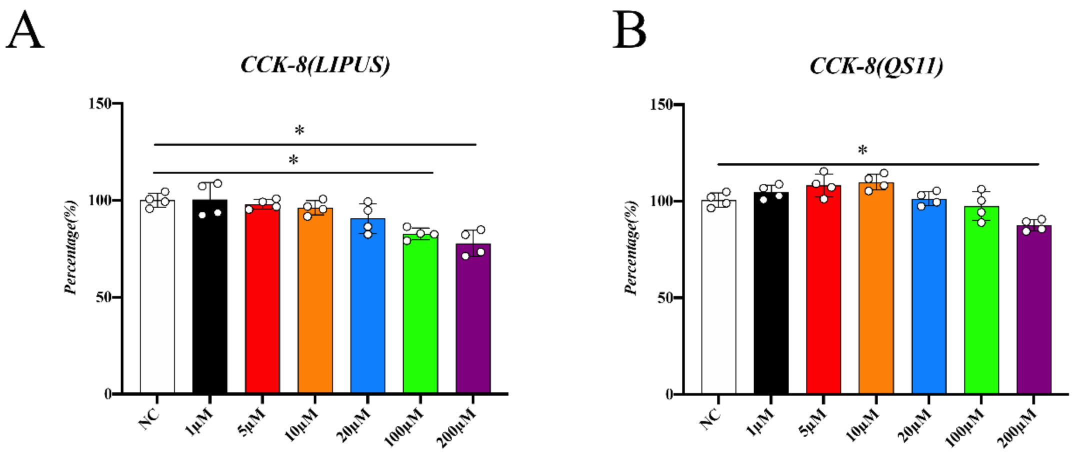

S10. Drug titration (XAV-939 and QS11) curve with cell viability(RAW264.7).

(A-B) Cell Counting Kit-8 (CCK-8) is tested at different concentration of XAV-939 and QS11 for cell viability of RAW264.7. (n=4) *P < 0.05. **P < 0.01.

Supplemental Movies

Sup M1. Ultrasound therapy in mice

Sup M2. Water maze test

Sup M3. Catwalk

Sup M4. Rotarod test

Sup M5. Treadmill test

Sup M6. Ultrasound treatment in vitro

Original Western Blot Date

Related to Fig.4 (C) :
Day 3 In vivo

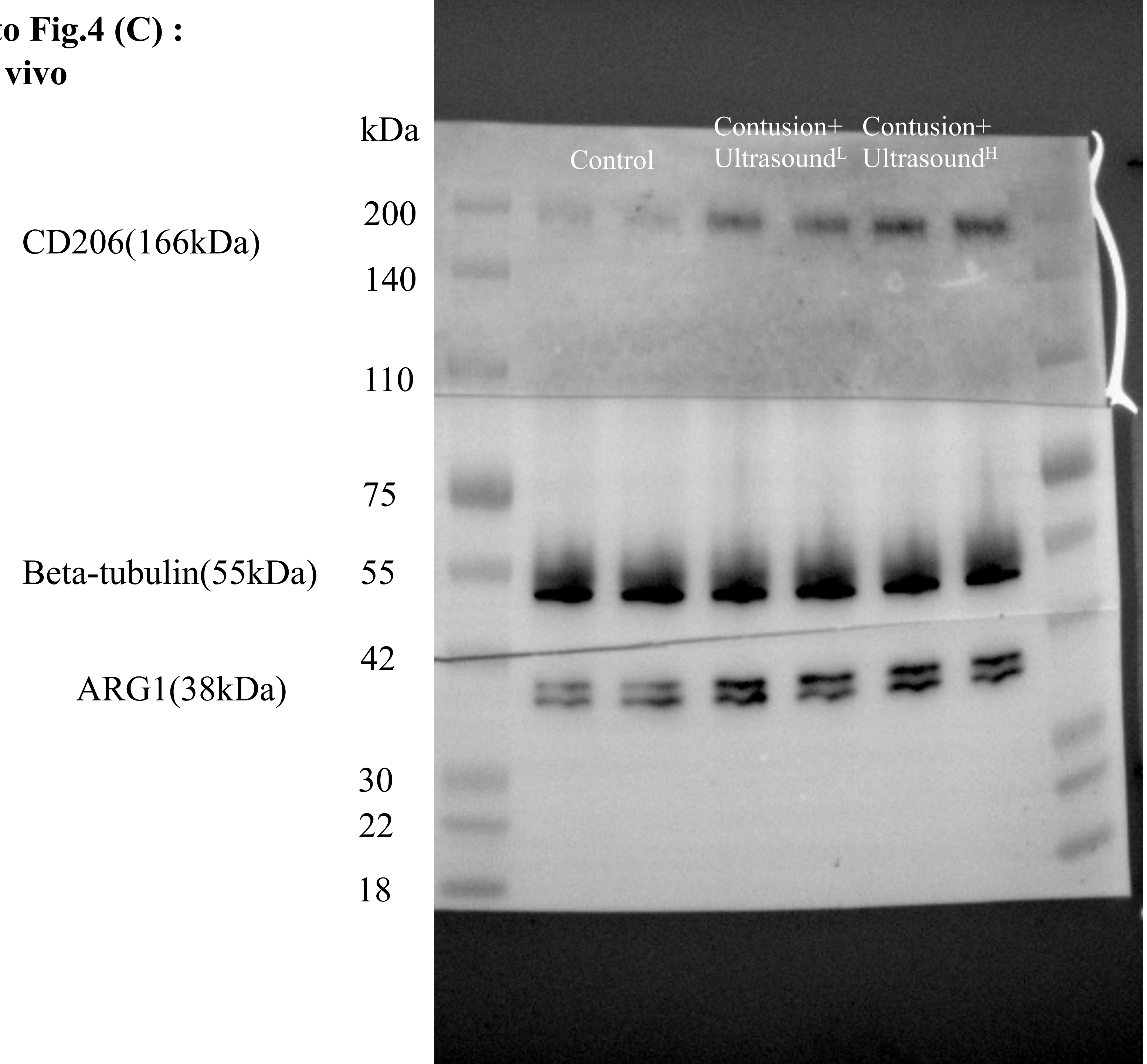

Related to Fig.4 (C):
Day 7 In vivo

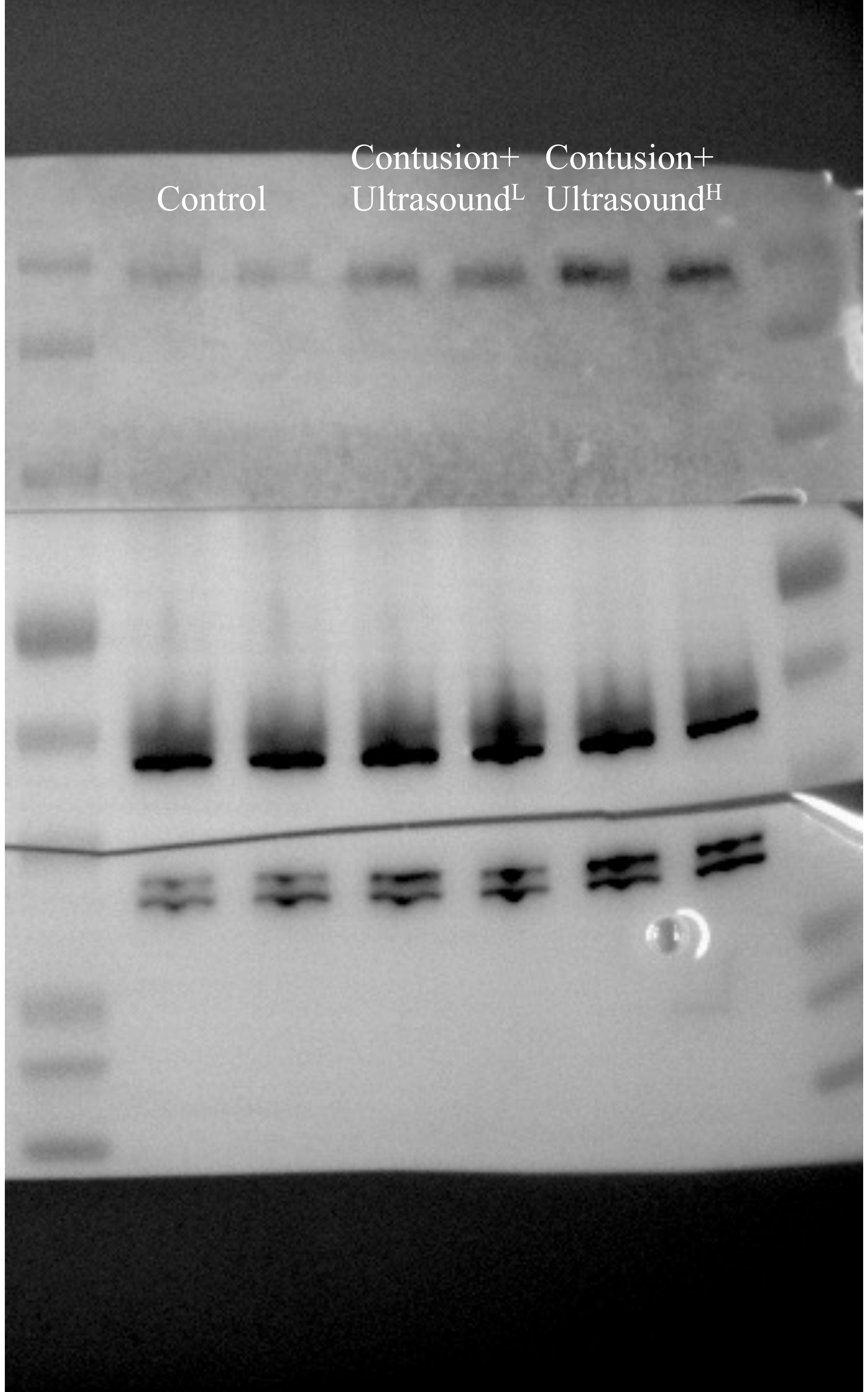

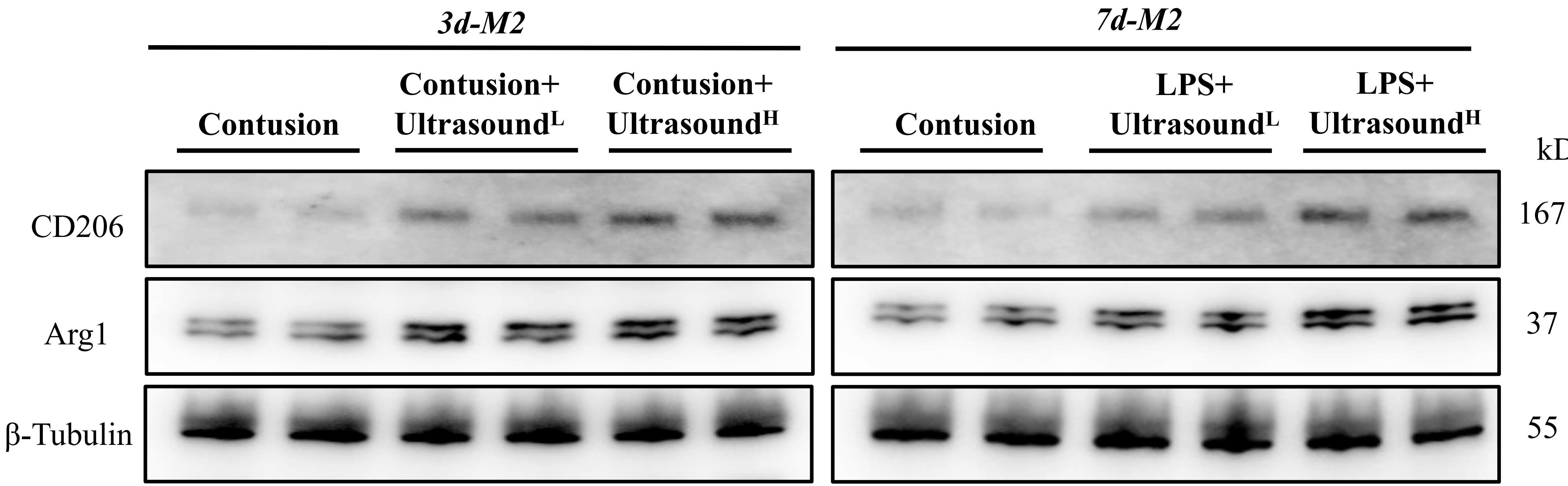
3d-M2
7d-M2
Contusion
Contusion+
UltrasoundL
Contusion+
UltrasoundH
Contusion
LPS+
UltrasoundL
LPS+
UltrasoundH
kD
CD206
Arg1
β-Tubulin
167
37
55

Related to Fig.4 (c):
Day 3 In vivo

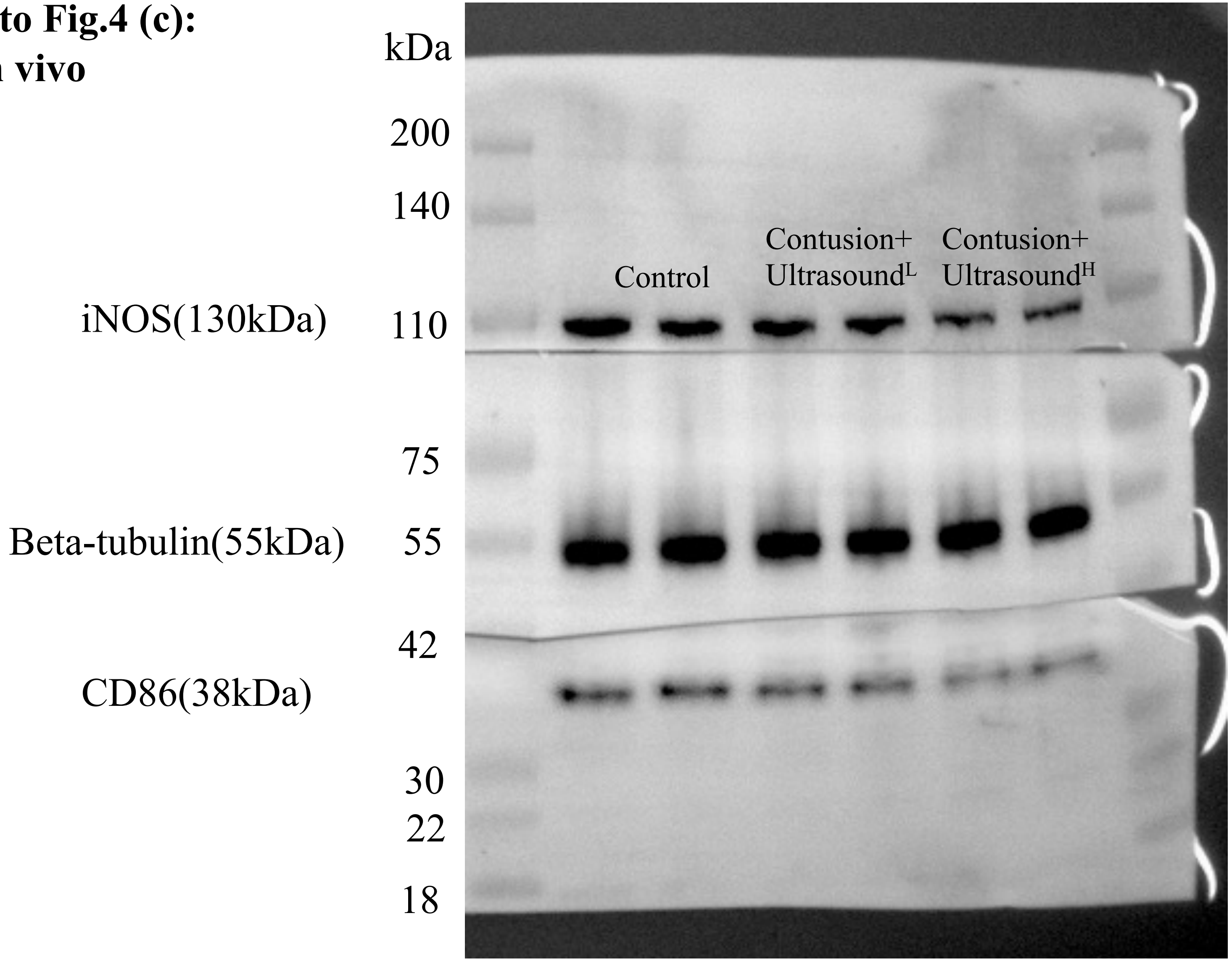

Related to Fig.4 (c):
Day 7 In vivo

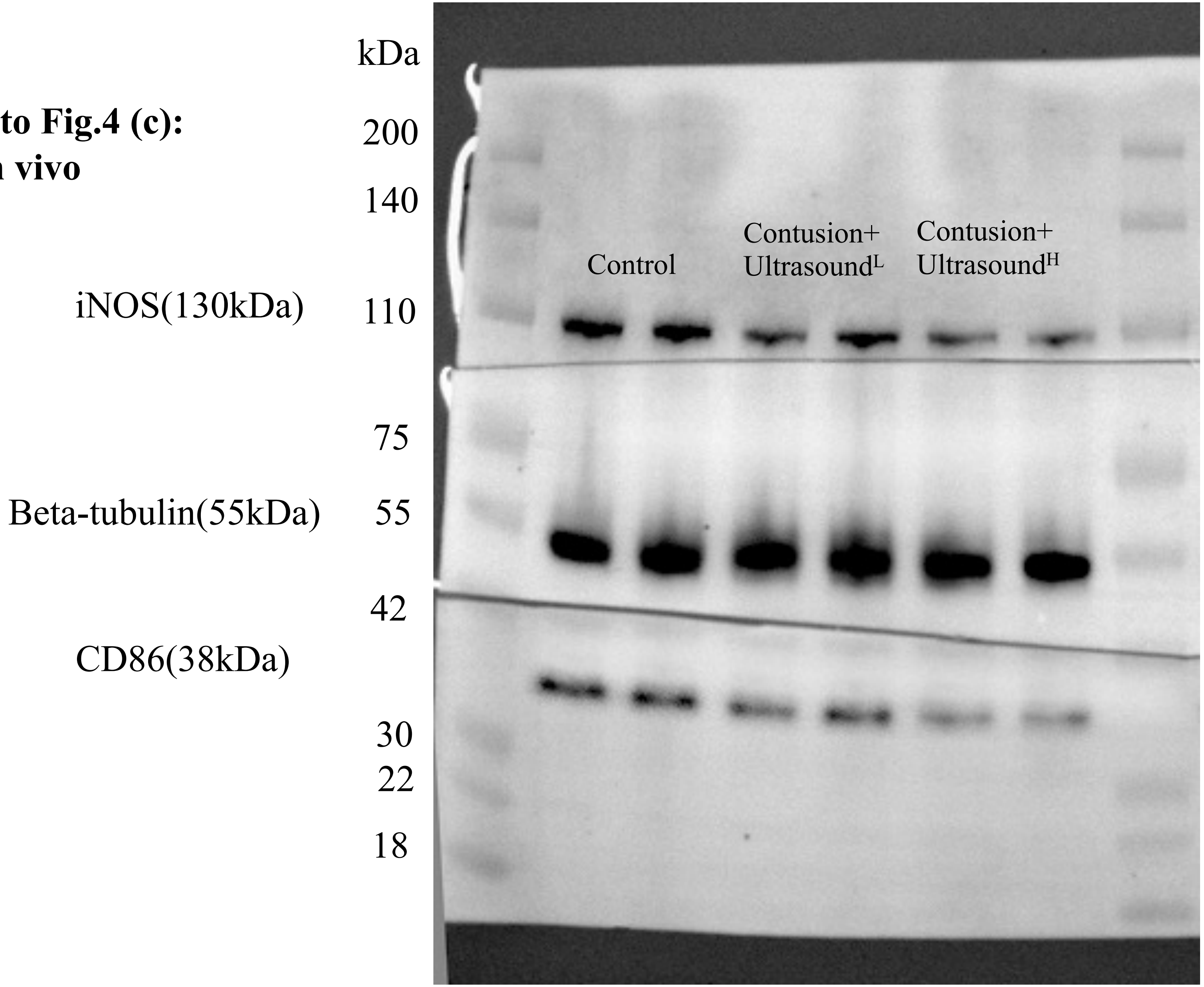

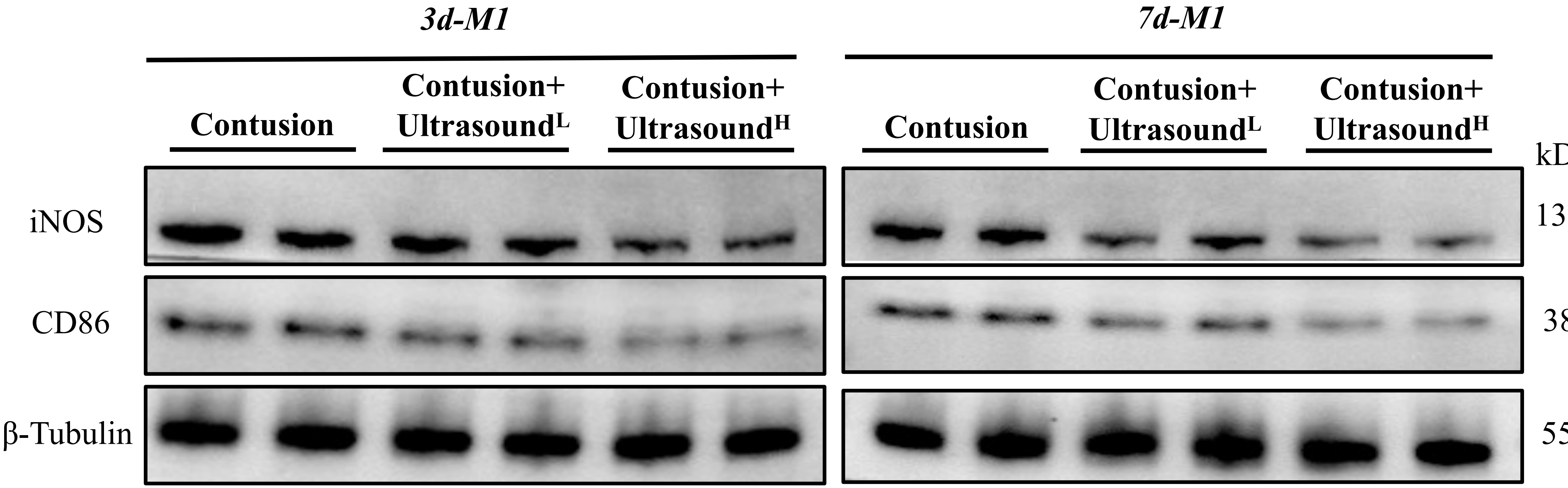
3d-M1
7d-M1
Contusion
Contusion+
Ultrasound^L
Contusion+
Ultrasound^H
Contusion
Contusion+
Ultrasound^L
Contusion+
Ultrasound^H
kD
iNOS
13
CD86
38
β-Tubulin
55

Related to Fig.6 (E):
In vitro

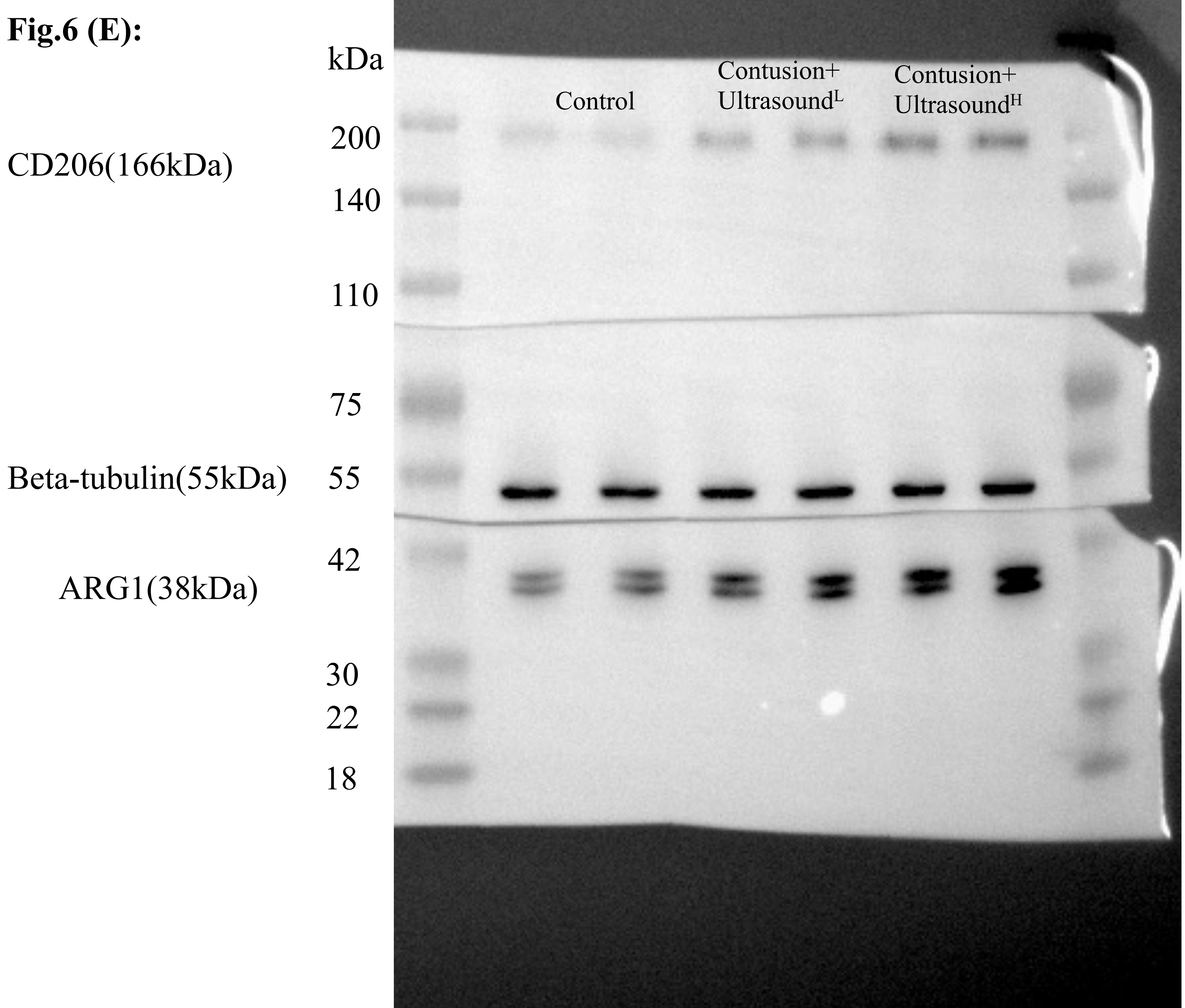

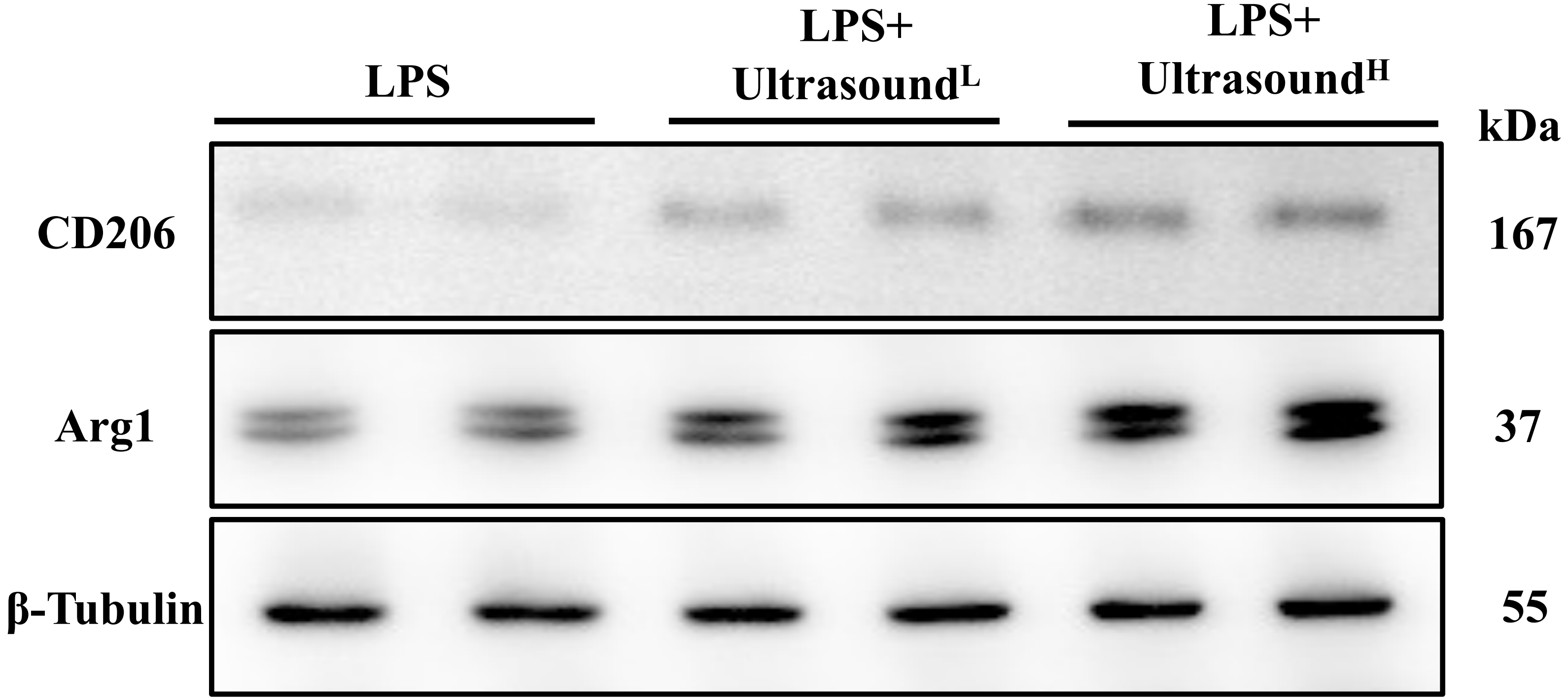

LPS
LPS+
Ultrasound^L
LPS+
Ultrasound^H
kDa
CD206
167
Arg1
37
β-Tubulin
55

Related to Fig.6 (E):
In vitro

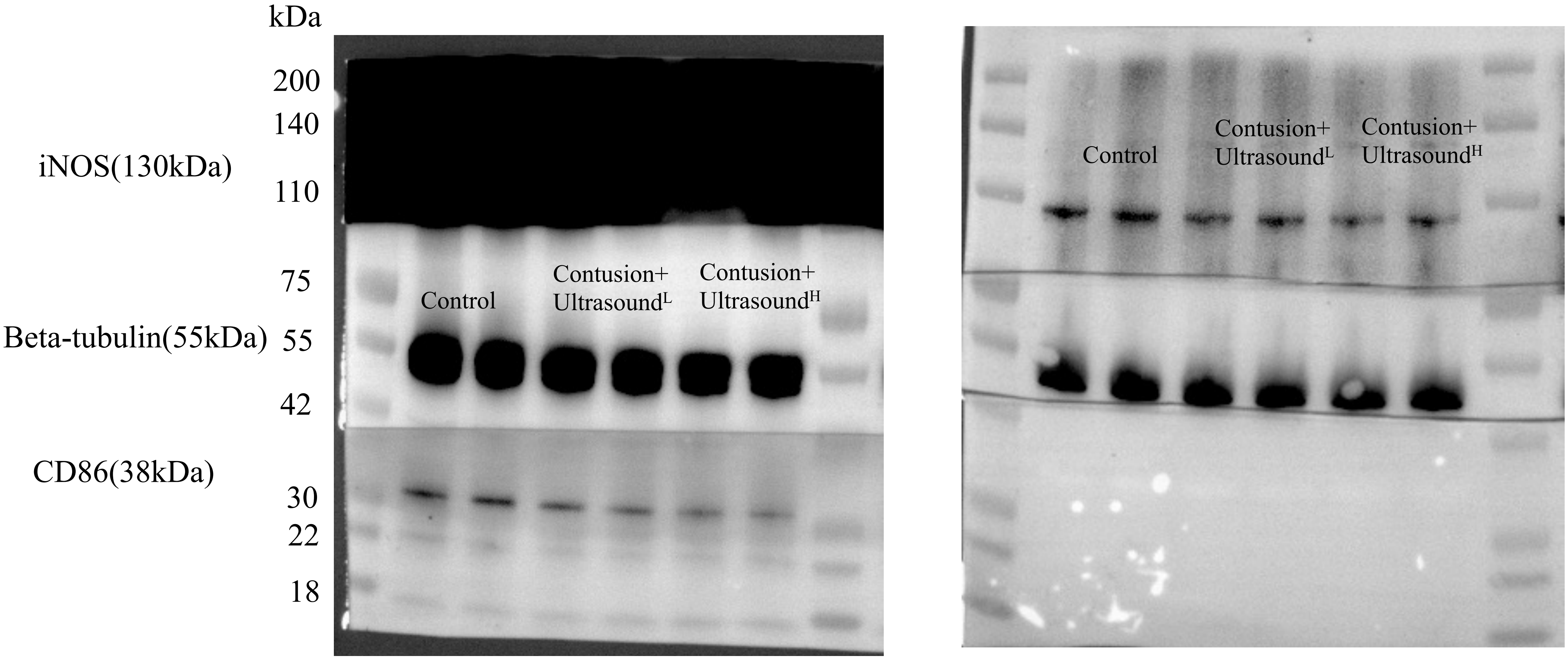

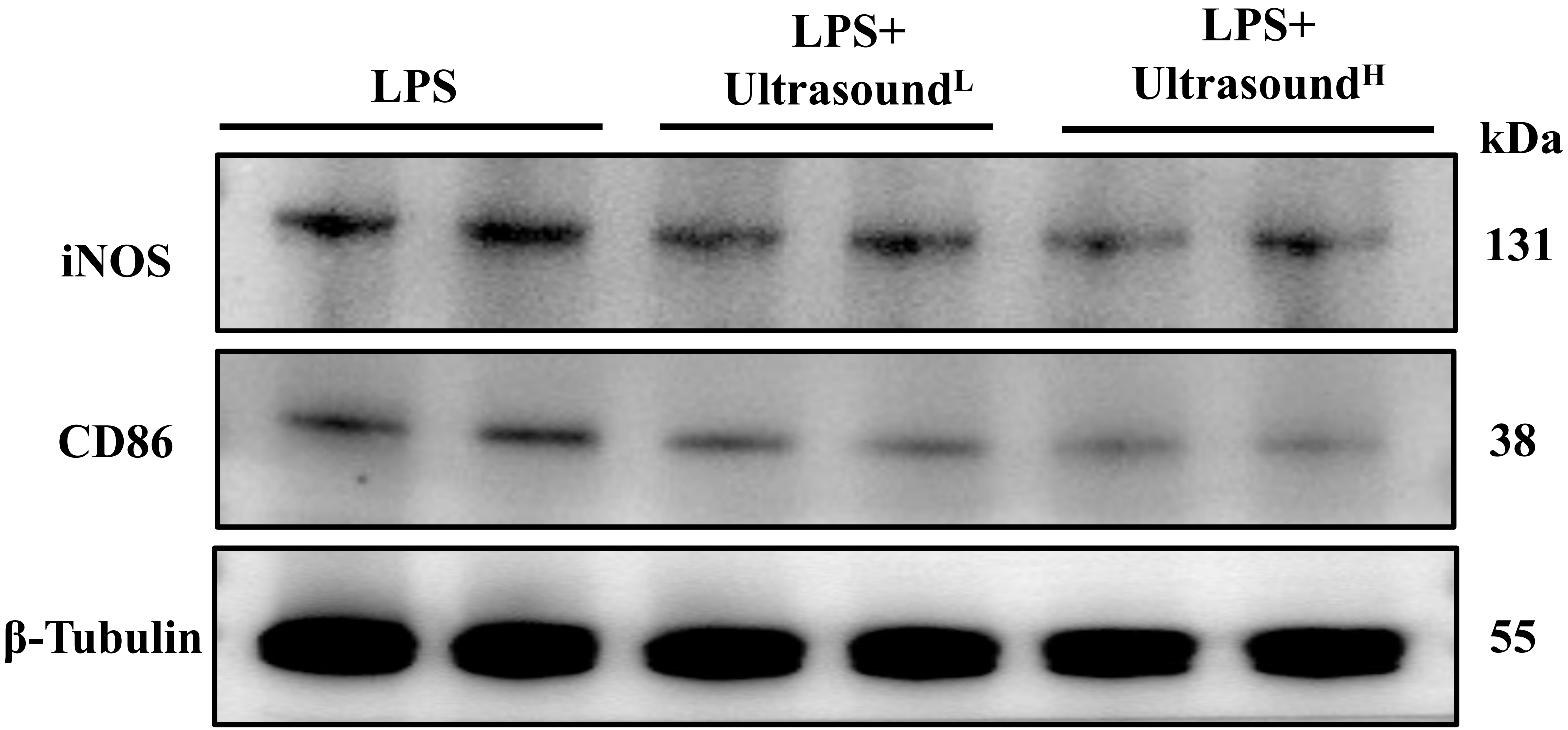
LPS
LPS+
Ultrasound^L
LPS+
Ultrasound^H
kDa
iNOS
131
CD86
38
β-Tubulin
55

Related to Fig.7 (A):
In vitro

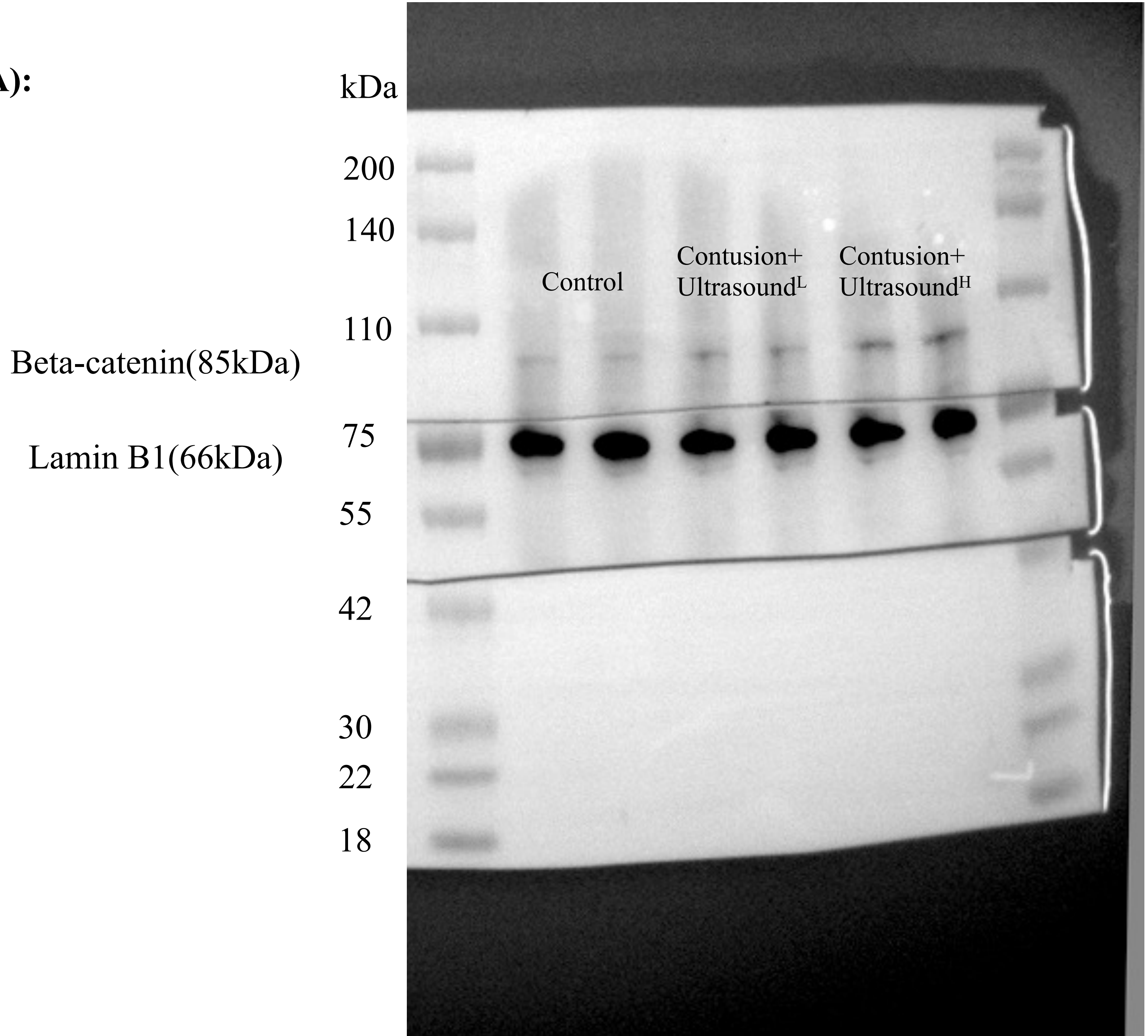

Related to Fig.7 (A):
In vitro

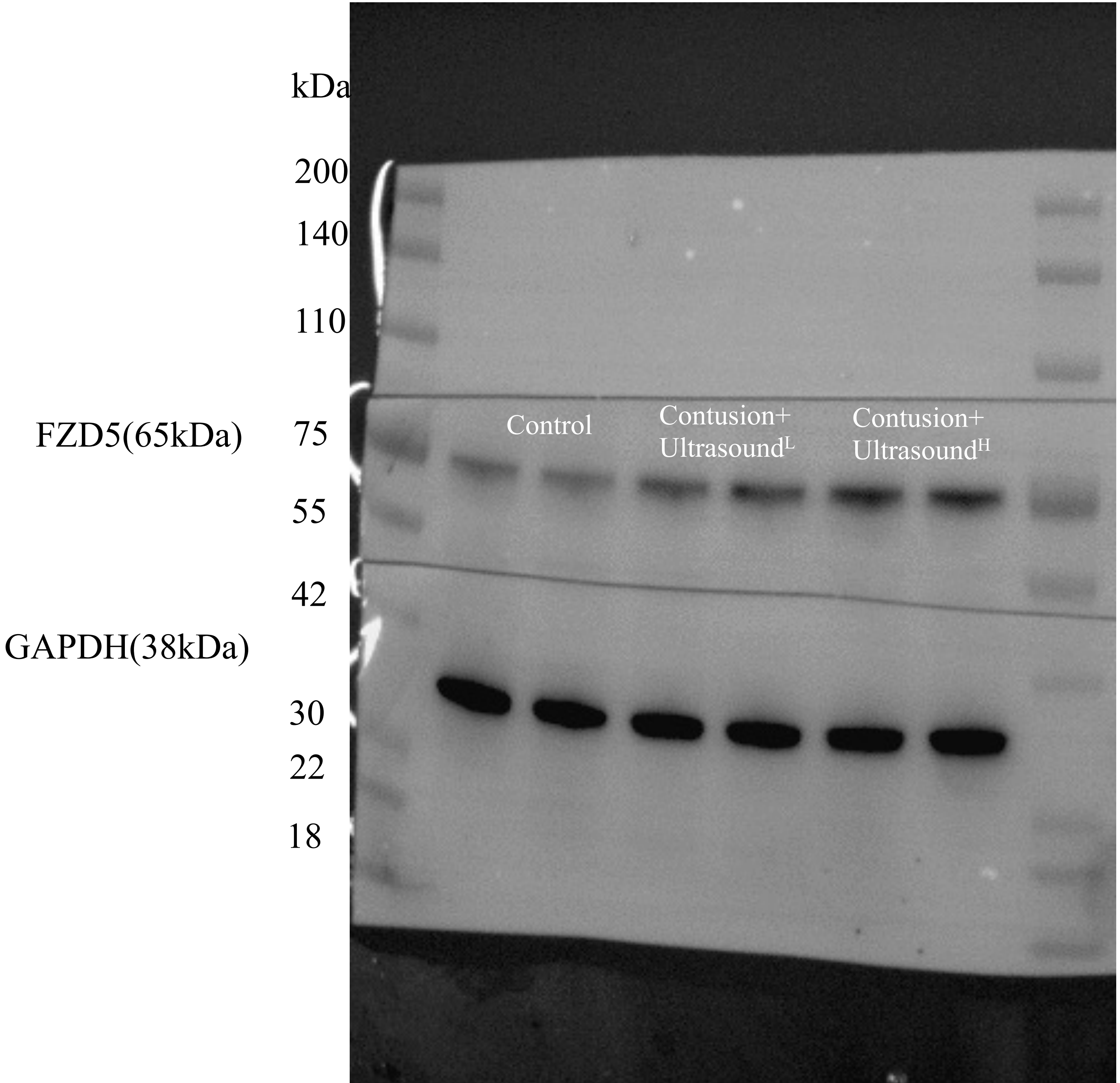

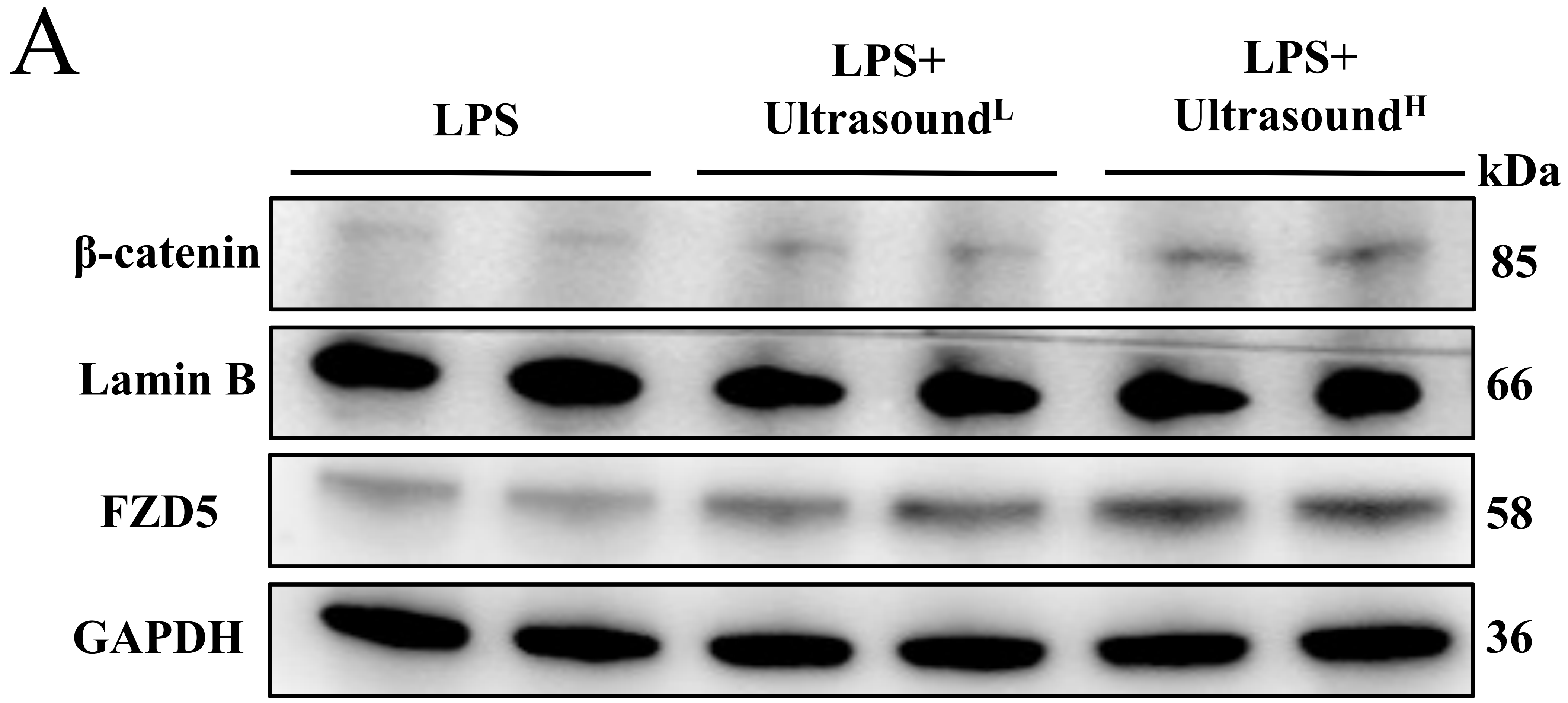
A
LPS
LPS+
Ultrasound^L
LPS+
Ultrasound^H
kDa
β-catenin
85
Lamin B
66
FZD5
58
GAPDH
36

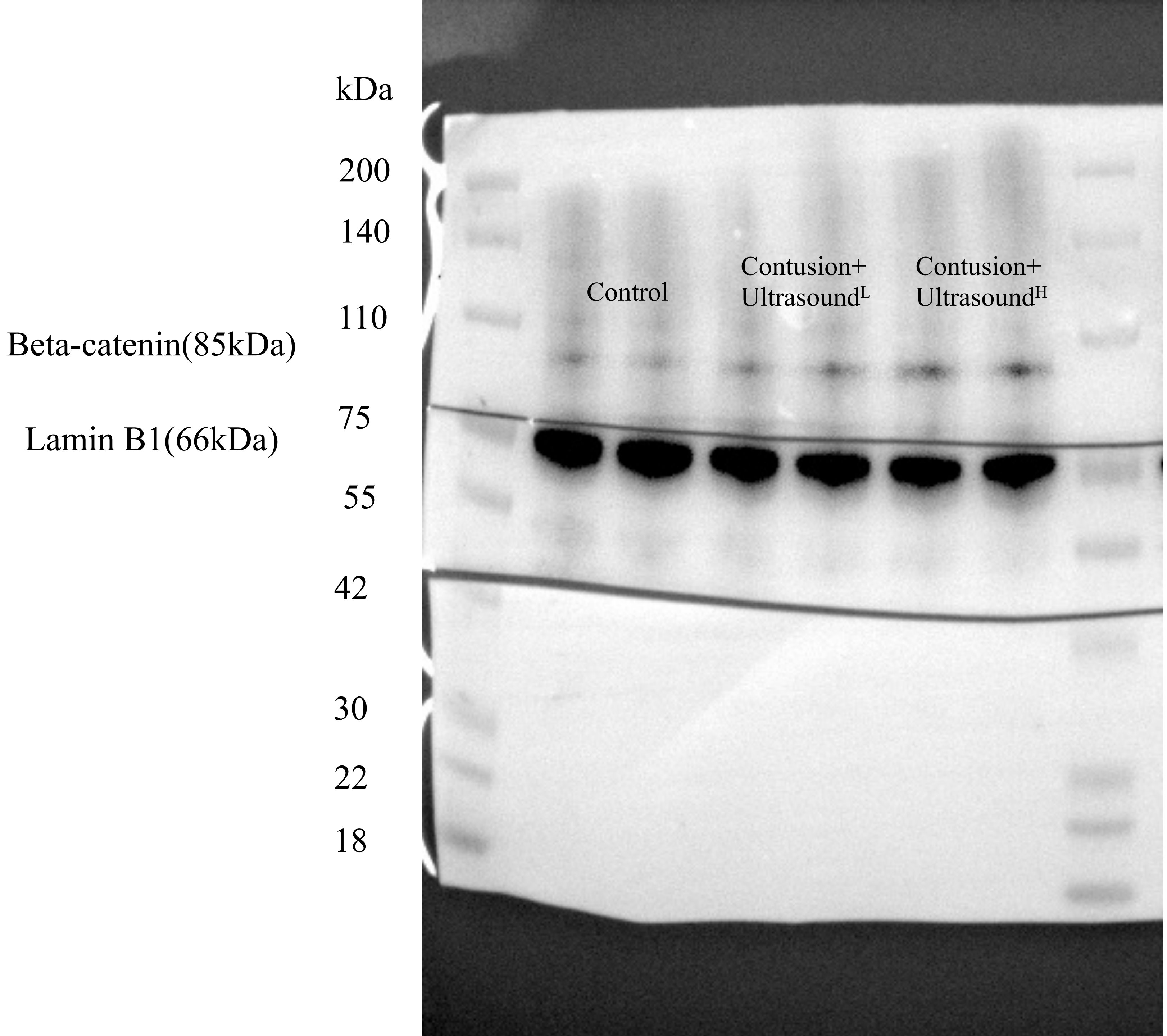
kDa
200
140
110
75
55
42
30
22
18
Beta-catenin(85kDa)
Lamin B1(66kDa)
Control
Contusion+
Ultrasound^L
Contusion+
Ultrasound^H

Related to Fig.7 (A):
In vivo

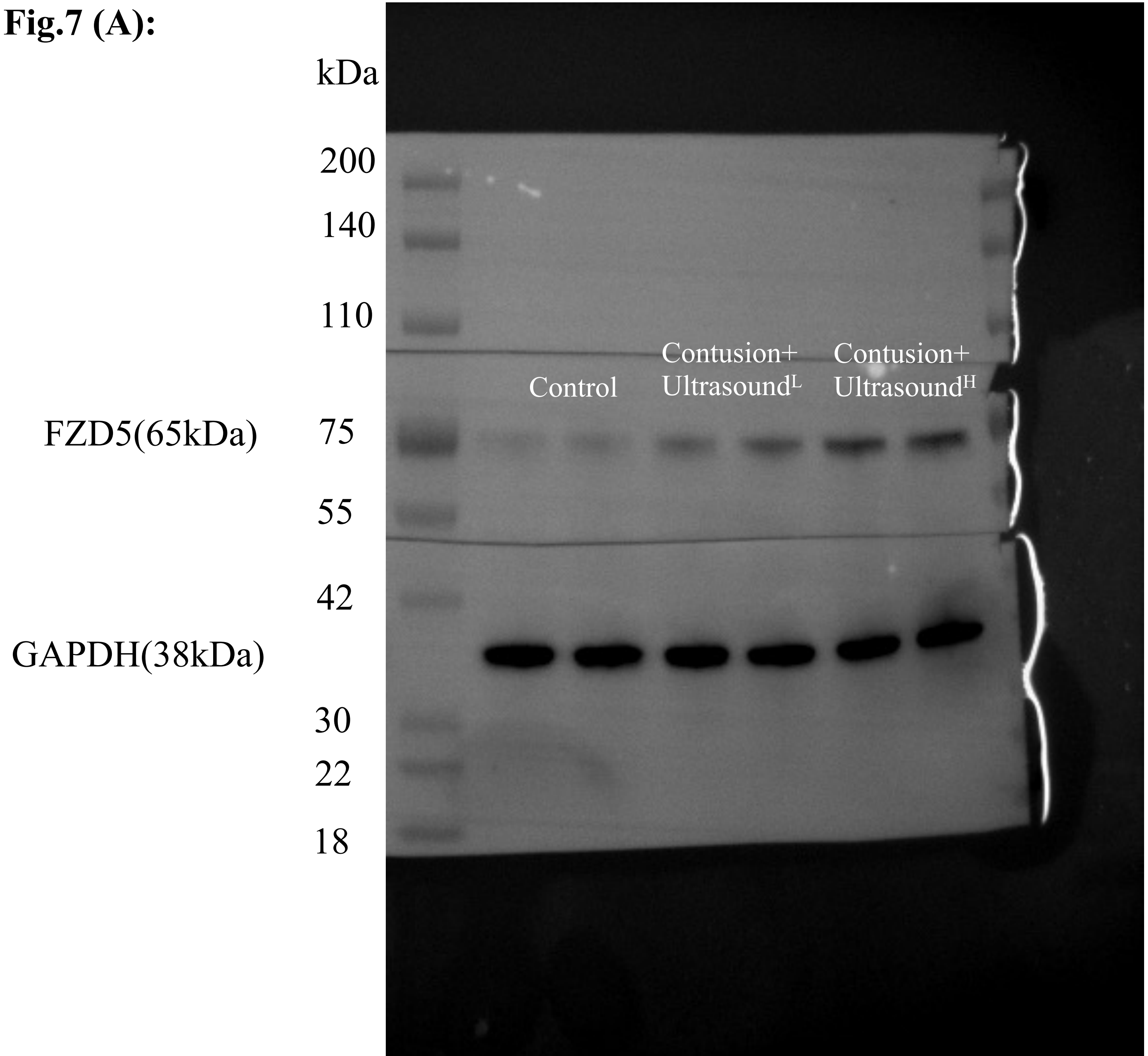

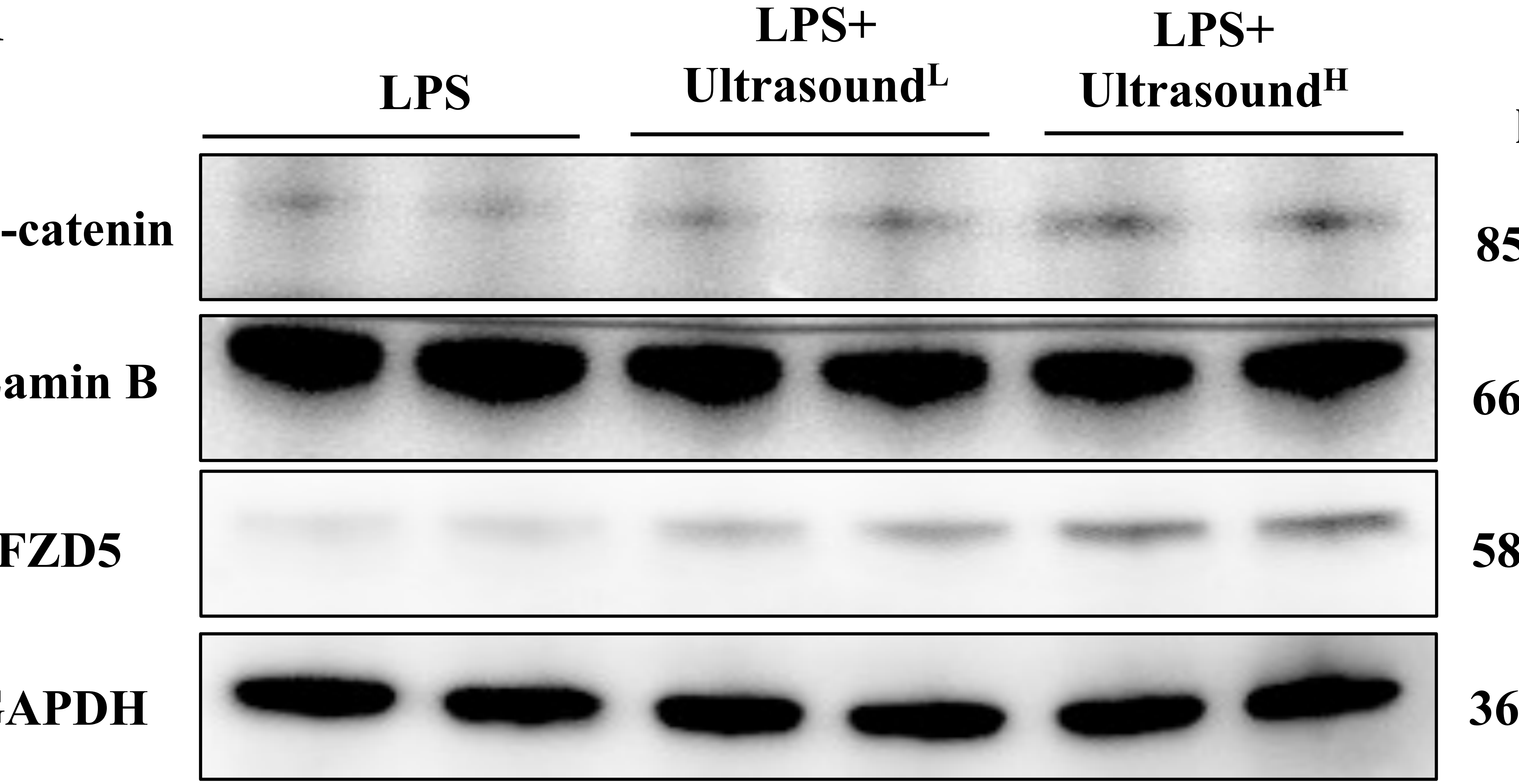

LPS
LPS+
Ultrasound^{L}
LPS+
Ultrasound^{H}
-catenin
amin B
FZD5
GAPDH
85
66
58
36

Supplemental Movies
Sup M1. Ultrasound therapy in mice
Sup M2. Water maze test
Sup M3. Catwalk
Sup M4. Rotarod test
Sup M5. Treadmill test
Sup M6. Ultrasound treatment in vitro
